# Peptide/β-Peptoid Hybrids with Ultrashort PEG-Like Moieties: Effects on Hydrophobicity, Antibacterial Activity and Hemolytic Properties

**DOI:** 10.3390/ijms22137041

**Published:** 2021-06-30

**Authors:** Nicki Frederiksen, Stavroula Louka, Chirag Mudaliar, Ilona Domraceva, Agrita Kreicberga, Osvalds Pugovics, Dorota Żabicka, Magdalena Tomczak, Weronika Wygoda, Fredrik Björkling, Henrik Franzyk

**Affiliations:** 1 Center for Peptide-Based Antibiotics, Department of Drug Design and Pharmacology, Faculty of Health and Medical Sciences, University of Copenhagen, DK-2100 Copenhagen, Denmark; nicki.frederiksen@sund.ku.dk (N.F.); stavroulaloukad@gmail.com (S.L.); chiragmudaliar@gmail.com (C.M.); fb@sund.ku.dk (F.B.); 2 Latvian Institute of Organic Synthesis, Aizkraukles 21, 1006 Riga, Latvia; ilona@farm.osi.lv (I.D.); agrita.kreicberga@osi.lv (A.K.); osvalds@osi.lv (O.P.); 3 Department of Epidemiology and Clinical Microbiology, National Medicines Institute, ul. Chełmska 30/34, 00-725 Warsaw, Poland; d.zabicka@nil.gov.pl (D.Ż.); mtomczak@cls.edu.pl (M.T.); w.wygoda@nil.gov.pl (W.W.)

**Keywords:** peptidomimetics, ultrashort PEG-like moieties, hydrophobicity, antibacterial activity, hemolysis, HepG2 cell viability

## Abstract

PEGylation of antimicrobial peptides as a shielding tool that increases stability toward proteolytic degradation typically leads to concomitant loss of activity, whereas incorporation of ultrashort PEG-like amino acids (sPEGs) remains essentially unexplored. Here, modification of a peptide/β-peptoid hybrid with sPEGs was examined with respect to influence on hydrophobicity, antibacterial activity and effect on viability of mammalian cells for a set of 18 oligomers. Intriguingly, the degree of sPEG modification did not significantly affect hydrophobicity as measured by retention in reverse-phase HPLC. Antibacterial activity against both wild-type and drug-resistant strains of *Escherichia coli* and *Acinetobacter baumannii* (both Gram-negative pathogens) was retained or slightly improved (MICs in the range 2–16 µg/mL equal to 0.7–5.2 µM). All compounds in the series exhibited less than 10% hemolysis at 400 µg/mL. While the number of sPEG moieties appeared not to be clearly correlated with hemolytic activity, a trend toward slightly increased hemolytic activity was observed for analogues displaying the longest sPEGs. In contrast, within a subseries the viability of HepG2 liver cells was least affected by analogues displaying the longer sPEGs (with IC_50_ values of ~1280 µg/mL) as compared to most other analogues and the parent peptidomimetic (IC_50_ values in the range 330–800 µg/mL).

## 1. Introduction

Increased prevalence of multidrug-resistant (MDR) pathogens constitutes an escalating worldwide problem [1]. Continued excessive use and misuse of antibiotics contribute to the development of antimicrobial resistance (AMR) in bacteria, thereby creating a serious health threat. Traditional strategies for introduction of new antibiotics into the clinic have not been successful in recent decades. In particular, it is critical to advance development of novel antibacterials different from those arising from modification of existing classes of antibiotics or screening of compound libraries [2]. From 2000 to October 2019, only five out of 38 (13%) new antibacterial drugs launched worldwide were first-in-class [2]. In contrast, 37% of all new drugs approved by the U. S. Food & Drug Administration (FDA) in the same period were first-in-class [3]. Hence, antibacterial agents with novel modes of action are required to combat pathogens possessing AMR against last-resort antibiotics [4,5,6,7]. To alleviate this situation it is urgent to explore non-traditional leads for new effective antibacterials.

Antimicrobial peptides (AMPs) constitute a source of potential antibiotics with modes of action differing from those of most classical antibiotics. Several AMPs have been approved by the FDA [8,9], but the inherently low in vivo stability of peptides restricts their clinical applications [10,11]. This limitation has urged the development of synthetic AMPs, of which none has entered the market so far, albeit several are currently undergoing clinical trials [8]. Furthermore, many classes of stable peptidomimetics are currently being investigated [12]. Peptide/peptoid hybrids constitute an interesting class of peptidomimetics, and among such oligomers with an alternating cationic–hydrophobic design we previously identified subclasses possessing a superior pharmacological profile as compared to the corresponding peptides and peptoid homooligomers [13]. In subsequent studies, peptide/peptoid hybrid oligomers were found to possess potent activity against Gram-negative pathogens, e.g., *Escherichia coli* [14,15,16,17], *Pseudomonas aeruginosa* [15,16,17,18], and *Acinetobacter baumannii* [16,18] as well as toward Gram-positive pathogens, e.g., *Staphylococcus aureus* [15,17,18] and *Enterococcus faecium* [18].

Modification of AMPs with polyethylene glycol (PEG) moieties at the N-terminus has been used as a strategy to overcome the challenge of low in vivo stability of AMPs [19,20]. The molecular weights (MWs) of PEG chains introduced to improve the bioavailability of AMPs and other biopharmaceuticals are typically high (i.e., 5–40 kDa [21]). While the extensive PEG chains effectively shield the AMP sterically from proteolytic degradation, they may also restrict the accessibility of its bioactive domain(s), thereby diminishing its biological activity [19,21]. Low-MW PEG chains (i.e., MW in the range 2–5 kDa) have similarly been applied, but even these may confer reduced potency [19]. Thus, two previous studies by Imura et al. showed that attachment of a 5 kDa PEG moiety at the N-terminus of tachyplesin I and magainin 2 resulted in conjugates with lowered cytotoxicity. While their modes of action essentially remained unchanged, both PEG-tachyplesin I and PEG-magainin 2 exhibited decreased antibacterial activity due to diminished binding affinity and destabilization of the secondary structure, respectively [22,23]. Similarly, Cui et al. showed that a PEGylated analogue of the AMP OM19r-8 (i.e., VDKPPYLPRPRPIRrPGGr-NH_2_), mPEG_5_-OM19r-8 (with a 5 kDa PEG), displayed a 2.5-fold lower antibacterial activity as compared to that of OM19r-8, but exhibited a significantly improved stability in the presence of e.g., increased temperature, trypsin, papain, 50% serum, or high salt concentration [20]. Additionally, the elimination half-life of mPEG_5_-OM19r-8 in rats was improved from 1.6 to 28 min. Retained antibacterial activity and increased protease stability was reported for the PEGylated AMPs CaLL [24], the melittin derivative MA [25], SET-M33L [26], and the tetrabranched AMP M33 [27]. Conversely, Grimsey et al. reported that PEGylation (with 2 kDa PEG) of the AMP A1 (i.e., RIRIRWIIR-NH_2_) abolished its antibacterial activity [28]. Furthermore, PEGylation with low-MW PEG chains has been used in prodrugs of AMPs and peptidomimetics, including apidaecin and oncocin [29,30], a short Arg-rich AMP derived from LL37 [31], and oligothioetheramides [32].

Both for AMPs and peptide/peptoid hybrid oligomers, correlations between relatively high hydrophobicity and increased antibacterial and hemolytic activity have been found [33,34,35,36]. Recently, we investigated an extended series of closely related peptidomimetics, and it was found that compounds with a hydrophobicity below a certain threshold (which depends on the bacterial species) were devoid of antibacterial activity, while compounds with a hydrophobicity exceeding a limiting threshold lacked cell selectivity [17]. This finding corroborates similar observations reported for peptoids and AMPs in earlier studies [16,37,38,39,40].

While partition coefficients, e.g., log D values, are typically utilized in the estimation of the hydrophobicity of small molecules, the relative retention in reverse-phase HPLC (RP-HPLC) represents a more appropriate measure of hydrophobicity for very polar and highly cationic peptides and peptidomimetics [17,36,41,42,43,44]. Recently, within a large set of peptidomimetics, resembling those in the present study, we identified a correlation between hydrophobicity (expressed as percent acetonitrile at peak elution in analytical RP-HPLC) and both antibacterial activity and hemolytic properties [17].

In the present study, we aimed at obtaining an improved understanding of how modification of antibacterial peptide/peptoid hybrids with ultrashort PEG-like residues (hereafter termed sPEGs) influence hydrophobicity and biological activity profiles of the resulting analogues. Thus, a set of 17 sPEG-containing hybrids and their parent oligomer were analyzed on four different RP-HPLC columns to determine their retention time. Furthermore, it was attempted to estimate log D values for a subset of compounds to allow for a comparison of the relative hydrophobicity as measured by these two methods.

Additionally, the present series of peptide/peptoid hybrid oligomers were tested against a panel comprising six bacterial strains, representing most ESKAPE bacteria (*E. faecalis*, *S. aureus*, *K. pneumoniae*, *A. baumannii*, *P. aeruginosa*) [45,46] and *E. coli*. Finally, the effects exerted by these compounds on human red blood cells (hRBC) and HepG2 liver cells were determined.

## 2. Results and Discussion

### 2.1. Selection and Synthesis of Peptidomimetics

**Figure 1:**
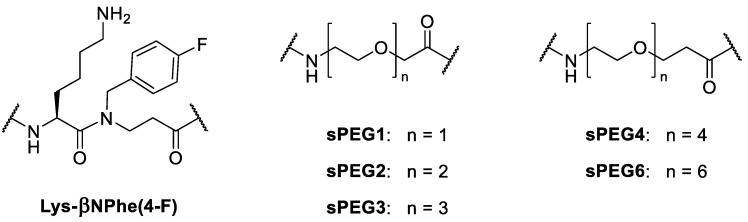
Structures of Lys-βNPhe(4-F) dimeric unit and ultrashort PEG-like amino acids (sPEGs).

A series of sPEG-containing oligomers were designed based on the previously studied 12-mer oligomer H-[Lys-βNPhe(4-F)]_6_-NH_2_, consisting of alternating L-lysine and 4-fluorophenylalanine-like β-peptoid units (i.e., *N*-(4-fluorobenzyl)-β-alanine, abbreviated βNPhe(4-F); Figure 1) [18]. To investigate the effect of sPEGs attached at the termini or inserted at various positions within the sequence, these moieties were introduced via commercially available amino acids containing varying repeats (n times) of oxyethylene units (i.e., sPEGn; Figure 1).

The parent compound (i.e., **1**; Table 1; reported previously [18]) constitutes the basis for a comparative estimation of the effect of sPEG incorporation. Most examples of PEGylated AMPs reported in the literature contain much longer PEG chains at the termini [19,20,21,22,23,24,25,26,27,28,29,30,31,32], and therefore four oligomers with sPEG moieties at either the N-terminus (Table 1: subgroup I, i.e., **2** and **3**) or at both termini (subgroup II, i.e., **4** and **5**) were included in the present set of analogues. Additionally, to study the effect of both the positioning of the sPEG spacer units in the oligomer sequence and the number of sPEG moieties introduced, we included subgroup designs in which the parent 12-mer was separated into either two or three fragments of six or four residues, respectively. These fragment-based oligomers constitute three subgroups (see Table 1): five oligomers consisting of two peptide/β-peptoid hexamers separated by an sPEG residue of varying size (i.e., subgroup III: **6**–**10**), four oligomers consisting of two peptide/β-peptoid hexamers separated by an sPEG of varying size and displaying end-terminal sPEG moieties (i.e., subgroup IV: **11**–**14**), and finally four oligomers consisting of three peptide/β-peptoid tetramers separated by two sPEGs of varying size (i.e., subgroup V: **15**–**18**).

These 18 oligomers were synthesized by using Fmoc-based solid-phase synthesis for assembly of commercial sPEG amino acids and a preformed Fmoc-Lys(Boc)-βNPhe(4-F)-OH dimeric building block, which was synthesized by an aza-Michael addition approach as previously described [47,48]. Oligomers were synthesized on a Rink amide polystyrene resin, followed by acidic cleavage from the resin and subsequent purification by preparative HPLC, which yielded the desired compounds as trifluoroacetic acid (TFA) salts.

### 2.2. Introduction of Ultrashort PEG-Like Moieties: Influence on Hydrophobicity

For each peptide/β-peptoid hybrid oligomer, the retention time in RP-HPLC was measured on four different columns (details shown in Table 2): Phenomenex Luna C18(2) column (recommended for hydrophobic compounds [49]), Phenomenex Luna Omega Polar C18 column (recommended for both very polar and non-polar compounds [50]) as well as Phenomenex Aeris Peptide XB-C18 [51] and Sigma-Aldrich Supelco Discovery BIO Wide pore C18 [52] columns, which both are recommended for analysis of peptides. The retention times of the compounds on each column were used to calculate the acetonitrile concentration at peak elution (denoted merely as % MeCN; Table 1). As expected, the % MeCN for each compound varied somewhat between the four columns.

**Table 1:** Hydrophobicity of oligomers as estimated by retention in analytical RP-HPLC.

Subgroup	Cmpd	Sequence ^1^	% MeCN at Elution ^2^			
			Luna	Luna Omega	Aeris Peptide	Supelco Discovery
	**1**	H-[X]_6_-NH_2_	41.6	41.9	39.4	41.7
I	**2**	H-sPEG2-[X]_6_-NH_2_	41.4	41.9	39.4	41.5
	**3**	H-sPEG4-[X]_6_-NH_2_	41.9	42.4	39.6	42.6
II	**4**	H-sPEG2-[X]_6_-sPEG2-NH_2_	41.3	42.1	39.2	41.4
	**5**	H-sPEG4-[X]_6_-sPEG4-NH_2_	42.2	43.2	40.0	42.5
III	**6**	H-[X]_3_-sPEG1-[X]_3_-NH_2_	41.2	41.6	39.2	41.4
	**7**	H-[X]_3_-sPEG2-[X]_3_-NH_2_	41.4	41.8	39.4	41.6
	**8**	H-[X]_3_-sPEG3-[X]_3_-NH_2_	41.7	42.1	39.6	41.9
	**9**	H-[X]_3_-sPEG4-[X]_3_-NH_2_	41.8	42.3	39.8	42.0
	**10**	H-[X]_3_-sPEG6-[X]_3_-NH_2_	42.3	42.8	40.3	42.5
IV	**11**	H-sPEG1-[X]_3_-sPEG1-[X]_3_-sPEG1-NH_2_	40.8	41.4	38.7	40.8
	**12**	H-sPEG2-[X]_3_-sPEG2-[X]_3_-sPEG2-NH_2_	41.3	42.0	39.2	41.3
	**13**	H-sPEG3-[X]_3_-sPEG3-[X]_3_-sPEG3-NH_2_	41.9	42.8	39.9	42.1
	**14**	H-sPEG4-[X]_3_-sPEG4-[X]_3_-sPEG4-NH_2_	42.4	43.5	40.4	42.5
V	**15**	H-[X]_2_-sPEG1-[X]_2_-sPEG1-[X]_2_-NH_2_	41.1	41.4	39.0	41.2
	**16**	H-[X]_2_-sPEG2-[X]_2_-sPEG2-[X]_2_-NH_2_	41.5	41.9	39.4	41.6
	**17**	H-[X]_2_-sPEG3-[X]_2_-sPEG3-[X]_2_-NH_2_	41.8	42.4	39.8	42.0
	**18**	H-[X]_2_-sPEG4-[X]_2_-sPEG4-[X]_2_-NH_2_	42.1	42.7	40.1	42.3

**^1^** X = Lys-βNPhe(4-F). **^2^** Experimental relative hydrophobicity measured as % MeCN at peak elution. RP-HPLC gradient: 0→60% B during 15 min. For column specifications, see Table 2.

Notably, all compounds were retained to a lesser degree on the Aeris column as compared to the other columns that, on the other hand, provided very similar retention values when using the same gradient elution (i.e., 0% B→60% B during 15 min). Within each subgroup, the same ranking of the members with respect to hydrophobicity was observed. Additionally, within each subgroup gradual elongation of the sPEG moiety conferred a minor, but consistent, increase in retention.

Overall, compound 11 appeared to be the least hydrophobic, as reflected in the lowest % MeCN, while compound 14 exhibited the highest average % MeCN (the exception being on the Discovery column, where 3 displayed a slightly higher % MeCN). Expectedly, the Luna Omega column provided a marginally longer retention for all analogues in accordance with its recommended application for very polar compounds. In contrast, the Aeris Peptide column consistently resulted in approx. 2% lower values for the % MeCN at peak elution. Despite the relatively large difference in pore size between the Luna and Supelco Discovery columns, the % MeCN values varied only slightly (typically less than 0.3% with 3 having the maximal deviation of 0.7% MeCN). Thus, to perform a comparison of hydrophobicity based on RP-HPLC retention, the choice of column appears not to be crucial, since the four columns included here provided very similar absolute values for % MeCN at peak elution, and most importantly almost identical relative ranking of the members within the entire compound set.

The Luna C18(2) column was chosen as the reference column, and all % MeCN values stated in the following refer to this column. The relative hydrophobicity of the analogues as measured with this column ranged from 40.8% MeCN (for peptidomimetic **11**) to 42.4% MeCN (for peptidomimetic **14**), which constitutes a surprisingly narrow hydrophobicity window, with the parent compound **1** (41.6% MeCN) exhibiting an average hydrophobicity among all compounds.

**Figure 2:**
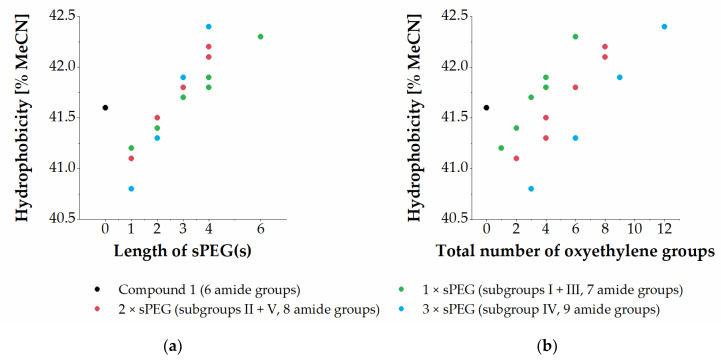
Relationship between the lengths of sPEG moieties incorporated (**a**) or total number of oxyethylene moieties incorporated (**b**) and hydrophobicity of the resulting compounds as measured by % MeCN at peak elution in RP-HPLC.

Half of the sPEG-containing analogues exhibited marginally increased hydrophobicity as compared to that of the parent compound **1**. Compounds containing sPEG1 and sPEG2 displayed decreased hydrophobicity as compared to **1**, while compounds containing sPEG3, sPEG4, or sPEG6 exhibited increased hydrophobicity when measured by their relative retention in RP-HPLC. Furthermore, a correlation between increased length of the sPEG moiety and increased hydrophobicity was observed within subgroups displaying the same number of amide bonds (see Figure 2a).

Comparison of oligomers across subgroups with varying content of a specific sPEG moiety showed that the hydrophobicity remained almost constant; thus **2**, **4**, **7**, **12** and **16** (i.e., sPEG2-containing) all have a hydrophobicity within the very narrow range of 41.3–41.5% MeCN, while **3**, **5**, **9**, **14** and **18** (i.e., sPEG4-containing) elute within 41.8–42.4% MeCN. Another way to analyse this compound set is to evaluate the effect on hydrophobicity conferred by the total number of oxyethylene moieties incorporated, which is seen to infer a weak, but consistent, trend toward increased hydrophobicity when raising the content of oxyethylene moieties (Figure 2b). Moreover, it is seen that within the mono-modified subgroups (i.e., I + III; green-colored in Figure 2b), bis-modified subgroups (i.e., II + V; red-colored), and tris-modified subgroup IV (blue-colored) there is a close-to-linear correlation between the number of oxyethylene groups and hydrophobicity.

When comparing sets of oligomers with the same total number of oxyethylene groups, but different total number of amide bonds, i.e., arising from sPEGs with different lengths, e.g., **3**, **4**, **9**, and **16** (all containing a total of four oxyethylene groups), and **10**, **12**, and **17** (all containing a total of six oxyethylene groups), a trend toward increased hydrophobicity was observed when extending the sPEG length, i.e., a single sPEG6 unit in **10** conferred higher hydrophobicity than three sPEG2 units in **12** (42.3% versus 41.3% MeCN). Thus, increased hydrophobicity was conferred by a higher total number of oxyethylene groups as well as by a single longer sPEG moiety over two or three shorter ones, i.e., a lower total number of amide groups. This infers that the content of amide bonds also is a factor contributing to the overall hydrophobicity, which most clearly is seen for the compounds containing a total of six oxyethylene groups, for which each additional amide bond reduces the hydrophobicity corresponding to 0.5% MeCN.

Additionally, we investigated the feasibility of both calculation and experimental determination of log D values to enable identification of a potential correlation with the biological activity of the present series of compounds. MarvinSketch has previously been employed for calculation of log D values of peptidomimetics [53]. Hence, this software was similarly utilized to calculate log D values for the present oligomers at pH 7.4 (*cLogD*_7.4_, see Table S2), which were in the range of −18.76 to −14.22 [54]. For a selected subset of the present series of compounds (i.e., **1**, **7**, and **9**–**16**) we also attempted to determine log D values (see Table S1). However, all these compounds proved to be exceedingly polar, and the limit of quantification of the applied LC–MS procedure was not low enough to allow for the determination of their actual concentration in the octanol phase. Instead, we measured indicative thresholds below which their log D values can be expected. Thus, based on the limits of quantification of the respective compounds all log D_max_ values were below −2.6. Hence, both calculation and direct determination of log D values was found to be hardly feasible for the members of the present class of peptidomimetics, potentially due to their polycationic and highly polar nature.

### 2.3. Introduction of sPEG Moieties: Influence on Antibacterial Activity

Peptide/β-peptoid hybrid oligomers were tested against a panel of six bacterial strains to determine their antibacterial activity (Table 3). The panel of wild-type strains comprised four Gram-negative species (*E. coli* ATCC 25922, *K. pneumoniae* ATCC 13883, *P. aeruginosa* PAO1, and *A. baumannii* ATCC 19606) and two Gram-positive pathogens (*S. aureus* ATCC 29213 and *E. faecalis* ATCC 29212). None of the compounds investigated in the present study exhibited activity against *K. pneumoniae* or *E. faecalis*. Additionally, the parent compound **1** exhibited moderate activity, as indicated by a minimal inhibitory concentration (MIC) of 16 µg/mL against *S. aureus*, while only analogues **2**, **6** and **16** (with 1–2 shorter sPEG moieties) had weak activity (i.e., MICs of 32–64 µg/mL) against this strain (Table 3).

Against *E. coli*, no significant change in antibacterial activity was observed in subgroups I and II (i.e., **2**–**5**) relative to that of the parent compound **1**, since all analogues exhibited similar or slightly decreased antibacterial activity (MICs in the range 8–16 µg/mL; Table 3). All subgroup III analogues (i.e., **6**–**10**) displayed similar antibacterial activity (MICs in the range 4–8 µg/mL) against *E. coli* as compared to that of **1**. Oligomers in subgroup IV (i.e., **11**–**14**) displayed similar activity (MICs in the range 8–16 µg/mL) as the subgroup I and II members (i.e., **2**–**5**). Finally, subgroup V compounds (i.e., **15**–**18**) exhibited similar or slightly increased antibacterial activity (MIC values of 2–8 µg/mL) against *E. coli* as compared to that of **1**.

Thus, the most active compound against *E. coli* was **16**. However, no clear correlations between sPEG length, number of sPEG moieties, or positioning of these in the sequence, and antibacterial activity against *E. coli* were observed. The only trend apparent was that most subgroup II and IV members (i.e., **4**, **5**, **12**–**14**), displaying sPEGs at the termini, had a marginally decreased antibacterial activity (MIC values in the range 8–16 µg/mL). Thus, no analogues of **1** exhibited clearly improved antibacterial activity (defined as at least a 4-fold lowering of the MIC) against *E. coli*.

Against *P. aeruginosa*, several sPEG-containing analogues showed similar antibacterial activity as compared to that of **1** (i.e., MICs of 16–64 µg/mL versus 32–64 µg/mL), while other analogues were devoid of activity (i.e., MIC > 64 µg/mL). No significant change in activity as compared to that of **1** was observed for subgroup I. In subgroup II **4** had slightly increased antibacterial activity (MIC of 16 µg/mL), while **5** was devoid of activity (i.e., MIC > 64 µg/mL). All compounds in subgroup III showed similar or slightly increased antibacterial activity against *P. aeruginosa* (MICs in the range 16–32 µg/mL). In subgroups IV and V, analogues containing the shorter sPEG1 and sPEG2 displayed similar activity as that of **1**, whereas analogues containing the elongated sPEG3 and sPEG4 lacked activity against *P. aeruginosa*.

Finally, against *A. baumannii*, several analogues displayed increased antibacterial activity as compared to that of **1**. Within subgroups I and II, **2**–**4** (containing 1–2 sPEG2 residues or a single sPEG4) typically exhibited 2-fold increased activity (i.e., MICs of 4–8 µg/mL) as compared to that of **1**, while **5,** containing two sPEG4 moieties, was equipotent to **1** (i.e., a MIC of 16 µg/mL). In subgroup III, all compounds exhibited approx. 2-fold increased potency (i.e., MICs in the range 4–8 µg/mL) toward *A. baumannii* as compared to that of **1**. The last two subgroups (IV and V) had very similar activity with MICs in the range 4–32 µg/mL as compared to the MIC of 8–16 µg/mL found for **1**.

**Figure 3:**
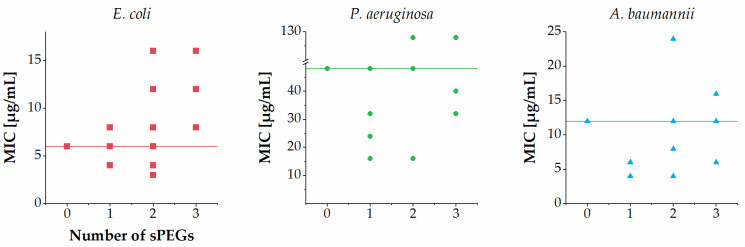
Relationship between number of sPEG residues and MIC against *E. coli*, *P. aeruginosa* and *A. baumannii*. MIC values determined as ranges are shown as the median value in the range (e.g., 4–8 µg/mL is shown as 6 µg/mL), while MIC values of >64 µg/mL are shown as 128 µg/mL. Horizontal lines refer to the MIC values of compound **1**.

**Figure 4:**
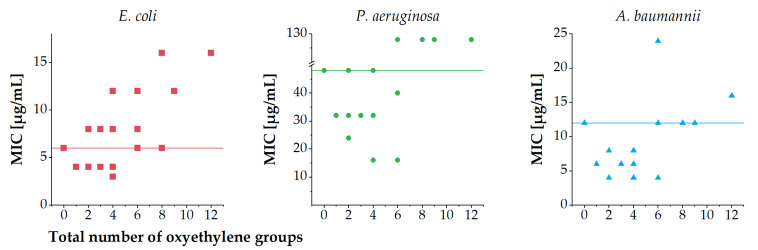
Relationship between total number of oxyethylene groups incorporated and MIC against *E. coli*, *P. aeruginosa* and *A. baumannii*. MIC values determined as ranges are shown as the median value in the range (e.g., 4–8 µg/mL is shown as 6 µg/mL), while MIC values of >64 µg/mL are shown as 128 µg/mL. Horizontal lines refer to the MIC values of the parent compound **1**.

Overall, the number of sPEGs was not clearly correlated with the MICs against *E. coli*, *P. aeruginosa* and *A. baumanni* (Figure 3). Further, a weak tendency toward a slightly lowered potency against *E. coli*, *P. aeruginosa* and *A. baumannii* was observed for members displaying larger sPEGs in subgroups II, IV and V. Across the entire series of analogues another trend was observed when considering the total number of oxyethylene groups incorporated (Figure 4): almost all analogues containing a total of one to four oxyethylene groups (i.e., **2**–**4**, **6**–**9**, **11**, **15**, and **16**) exhibited similar or slightly increased antibacterial activity against *E. coli*, *P. aeruginosa* and *A. baumannii* as compared to that of **1**. Conversely, almost all analogues containing a total of six or more oxyethylene groups (i.e., **5**, **10**, **12**–**14**, **17**, and **18**) exhibited similar or slightly decreased antibacterial activity against *E. coli*, *P. aeruginosa* and *A. baumannii* as compared to that of **1**. One exception to this trend was the sPEG6-containing **10**, which displayed slightly increased activity against both *P. aeruginosa* and *A. baumannii* as compared to that of **1**.

**Figure 5:**
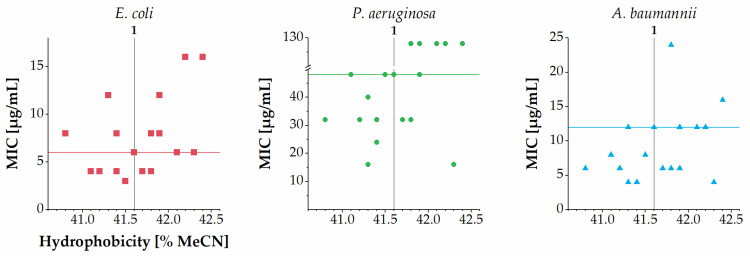
Relationship between hydrophobicity (as measured by % MeCN at RP-HPLC elution; hydrophobicity of the parent compound **1** is indicated by a vertical line) and MIC against *E. coli*, *P. aeruginosa* and *A. baumannii*. MIC values determined as ranges are shown as the median value in the range (e.g., 4–8 µg/mL is shown as 6 µg/mL), while MIC values of >64 µg/mL are shown as 128 µg/mL. Horizontal lines refer to the MIC values of the parent compound **1**.

Noticeably, the total number of oxyethylene groups and hydrophobicity (as measured by RP-HPLC) appeared to be positively correlated (Figure 2b). Although previous studies have found a trend of higher hydrophobicity conferring increased antibacterial activity against *P. aeruginosa* [16,17], several of the present sPEG-modified hybrid peptidomimetics deviate from this trend (Figure 5). Likewise, for *E. coli* and *A. baumannii* there seems to be no clear relationship between hydrophobicity and antibacterial activity for the present peptidomimetics. Different subclasses of peptide/peptoid hybrid peptidomimetics have been examined similarly, and the present findings for *E. coli* are in accordance with those earlier results, since no clear correlations between hydrophobicity and antibacterial activity against *E. coli* were found within any of the series of peptide/α-peptoid and peptide/β-peptoid oligomers investigated [16,17,18]. In contrast, for the most diverse set of peptide/α-peptoid hybrid oligomers, representing a wide range of hydrophobicity, a clear correlation between high hydrophobicity and potent antibacterial activity against *A. baumannii* was found [17]. In addition, in this respect, the present sPEG-modified peptidomimetics appear to be diverging, since most analogues have MIC values within a narrow range (i.e., 4–16 µg/mL) independently of their hydrophobicity (Figure 5). Nevertheless, it should be noted that the apparent absence of such clear correlations well may arise from the fact that all the sPEG-modified peptidomimetics studied here display remarkably small variations in hydrophobicity.

Previous studies of peptide/α-peptoid hybrid oligomers, covering a wide range of hydrophobicity, allowed identification of hydrophobicity thresholds below which compounds exhibited no antibacterial activity, e.g., peak elution at 36.0% MeCN for *E. coli* and 43.9% MeCN for *S. aureus* and *E. faecalis* [17]. By contrast, analogs eluting above 44% MeCN were extremely hemolytic. While the present series of compounds (having a hydrophobicity within the narrow range 40.8–42.4% MeCN) comply with these thresholds, a recent study infers that such hydrophobicity thresholds may vary slightly among different structural classes [55].

#### Antibacterial Activity against MDR Pathogens

All peptidomimetics (i.e., **1**–**18**) were also tested for their antibacterial activity against a panel of four MDR bacteria possessing AMR to at least one class of antibiotics (Table 4). Overall, the effect of AMR on the susceptibility of MDR *E. coli* strains to the peptide/β-peptoid hybrids appeared to be limited. Thus, for the colistin-resistant (mcr1-positive) *E. coli* isolate (NMI 3898/15) the MIC values were typically 2-fold higher, whereas the carbapenem-resistant and NDM-1-positive *E. coli* isolate (NMI 3371/16) had very similar susceptibility to most analogues. Likewise, colistin resistance in *A. baumannii* did not appear to alter the susceptibility of this strain toward the peptidomimetics. Conversely, while most compounds exhibited low to moderate activity against WT *P. aeruginosa*, all analogues were inactive against the colistin-resistant *P. aeruginosa* strain. These observations are in accordance with previous findings for the effect of AMR on the susceptibility of Gram-negative bacteria to peptide/α-peptoid hybrid oligomers [17].

### 2.4. Influence of sPEG Modification on How Peptidomimetics Affect Mammalian Cell Viability

The potentially damaging effects of the peptidomimetics on the viability of mammalian cells were tested in human red blood cells (Table 5). All compounds in the series gave rise to less than 10% hemolysis (ranging from 2.4% for **2** to 9.4% for **17**) at 400 µg/mL, corresponding to a 50- to 100-fold higher concentration than the typical MICs against *E. coli* and *A. baumannii*. While the number of sPEG residues did not appear to be closely correlated with hemolytic activity, a trend toward lowered hemolytic activity was observed for analogues displaying shorter sPEG moieties (i.e., either sPEG1 or sPEG2) except for subgroup III (i.e., **6**–**10**) where no correlation was apparent. Furthermore, selected compounds (i.e., **1**, **6**–**10**, **12**, and **14**–**18**) were tested for their antiproliferative activity in a cell viability assay with HepG2 liver cells (Table 5). The parent compound **1** and subgroup IV analogues **12** and **14** displaying end-terminal sPEG moieties were those exerting the most pronounced effect on HepG2 cell viability (IC_50_ values in the range 332–464 µg/mL). Interestingly, fragment-based analogues with no end-terminal sPEG moeities (i.e., subgroup III and V) all displayed decreased effect on HepG2 cell viability. In subgroup III (i.e., **6**–**10**), no clear correlation between sPEG size and effect on HepG2 cell viability was observed, since all IC_50_ values were within the relatively narrow range of 600–800 µg/mL. However, in subgroup V, analogues containing longer sPEG residues (i.e., sPEG3 and sPEG4) displayed ca. 2-fold higher IC_50_ values of approx. 1280 µg/mL (the highest concentration tested), as compared to those having shorter sPEGs (i.e., sPEG1 and sPEG2) with IC_50_ values of 568 and 687 µg/mL (for **15** and **16**, respectively).

The selectivity index (SI) constitutes a convenient parameter for estimating the potential therapeutic utility of the present peptidomimetics. When calculating the SI as the ratio between the IC_50_ value for the detrimental effect on HepG2 cells and the MIC it was seen that the most promising analogues against *E. coli* were compounds **16** and **18** having upper SI limits in the range 300–350 (Table 5), while for *A. baumannii* the best lead was compound **10** (with a single long sPEG6 unit) with an SI of 200. Based on these observations, modification of peptide/β-peptoid hybrid oligomers by incorporation of one or two sPEG residues may confer increased cell selectivity, in particular when two relatively longer sPEG residues are incorporated.

## 3. Materials and Methods

### 3.1. General Information

Starting materials and solvents were acquired from commercial suppliers (Iris Biotech, Markredwitz, Germany; Fluorochem, Hadfield, UK; and Merck, Darmstadt, Germany) and used without further purification. Water used for analytical and preparative high-performance liquid chromatography (HPLC) was filtered through a 0.22-μm capsule filter using an Evoqua LaboStar Pro TWF UV system. Purity and retention time of each peptidomimetic were determined by analytical HPLC by using a Phenomenex Luna Omega Polar C18 column (150 × 4.6 mm; particle size: 3 μm; pore size: 100 Å) on a Shimadzu Prominence and Shimadzu Nexera system using an aqueous acetonitrile (MeCN, VWR, Søborg, Denmark) gradient with 0.1% trifluoroacetic acid (TFA, Iris Biotech, Markredwitz, Germany) added (eluent A: 5:95 MeCN–H_2_O + 0.1% TFA, eluent B: 95:5 MeCN–H_2_O + 0.1% TFA); a flow rate of 0.8 mL/min was used. For elution of peptidomimetics, a linear gradient of 0% to 60% B during 15 min was used with UV detection at λ = 220 nm. All tested compounds had a purity of at least 95%. Retention time of each peptidomimetic was also determined by using a Phenomenex Luna C18(2) column (150 × 4.6 mm; particle size: 3 μm; pore size: 100 Å), a Phenomenex Aeris Peptide XB-C18 column (150 × 4.6 mm; particle size: 3.6 μm; pore size: 100 Å), and a Sigma-Aldrich Supelco Discovery BIO Wide pore C18 column (150 × 4.6 mm, particle size: 3 µm; pore size: 300 Å). For each peptidomimetic, the percentage of MeCN at peak elution was calculated from the retention time by using the following formula:

(1) $$\%\mathrm{MeCN}\;=0.95\;\left(0.6\frac{R_{t}}{15\;\min}\right)+0.05\;\left(1-0.6\frac{R_{t}}{15\;\min}\right)$$

Preparative HPLC was performed by using a Phenomenex Luna C18(2) column (250 × 21.2 mm; particle size: 5 μm) on a Shimadzu Prominence system using the same eluents as for analytical HPLC. Elution was performed with a linear gradient of 0% to 40% B during 20 min at a flow rate of 20 mL/min with UV detection at λ = 220 nm. High-resolution mass spectrometry (HRMS) spectra were obtained by using a Bruker Solarix XR MS detector.

### 3.2. General Protocol for Manual Synthesis of Peptidomimetics

The peptide/β-peptoid building block Fmoc-Lys(Boc)-βNPhe(4-F)-OH was synthesized as previously reported [47]. Peptidomimetics were then prepared manually as previously described [17]. In brief, H-Rink-Amide polystyrene resin (PCAS BioMatrix Inc., Saint-Jean-sur-Richelieu, QC, Canada; loading 0.53 mmol/g, 0.1 mmol) and Teflon vessels (10 mL; fitted with a polypropylene filter) were used. Coupling conditions used for the Fmoc-protected peptide/β-peptoid building block: 3.0 equiv building block, 3.0 equiv *N*,*N*′-diisopropylcarbodiimide (DIC, Iris Biotech, Markredwitz, Germany), and 3.0 equiv ethyl (hydroxyimino)cyanoacetate (OxymaPure^®^, CEM, Matthews, NC, USA) (>1 h under shaking at 40 °C). Coupling conditions used for Fmoc-protected sPEG amino acid building blocks: 3.0 equiv building block, 3.0 equiv (benzotriazol-1-yloxy)trispyrrolidinophosphonium hexafluorophosphate (PyBOP, Fluorochem, Hadfield, UK), and 6.0 equiv *N*,*N*-diisopropylethylamine (16 h under shaking at rt, Iris Biotech, Markredwitz, Germany).

Fmoc deprotection conditions: 20% piperidine (Iris Biotech, Markredwitz, Germany) in DMF (VWR, Søborg, Denmark; 2 × 10 min, each time with 5 mL under shaking at rt). Washing conditions: DMF, MeOH, and CH_2_Cl_2_ (VWR, Søborg, Denmark; 3 × 3 min, each time with 5 mL under shaking at rt). Capping was applied after loading: Ac_2_O–DIPEA–NMP (Iris Biotech, Markredwitz, Germany and VWR, Søborg, Denmark) 1:2:3 (2 × 5 mL, each time for 10 min under shaking at rt). Cleavage and side-chain deprotection were performed simultaneously with TFA–CH_2_Cl_2_ (95:5; 2 × 1 h, each with 5 mL under shaking at rt). The filtrates were collected, and the resin was further eluted with TFA (2 mL) and CH_2_Cl_2_ (2 mL). The combined filtrates were concentrated in vacuo and then co-evaporated with toluene (VWR, Søborg, Denmark; 3 × 5 mL). The crude product was purified by preparative HPLC, and the appropriate fractions were concentrated in vacuo and lyophilized. Identity was verified by HRMS, and purity (>95%) was determined by analytical HPLC.

### 3.3. Estimation of Log D Values

A phosphate buffered saline (PBS) pH 7.4 was prepared and then saturated with *n*-octanol. Likewise, an *n*-octanol solution saturated with pH 7.4 PBS buffer was also prepared. Both solutions were shaken for 24 h, and then left for 24 h in a separating funnel to ensure complete separation of the two phases. This provided an aqueous phase (PBS buffer saturated with *n*-octanol) and an *n*-octanol phase (saturated with PBS buffer).

Samples for log D determination were prepared as following: All compounds were dissolved in PBS buffer at a concentration 4 mg/mL, which was used as stock solutions. PBS-saturated *n*-octanol (1.0 mL), *n*-octanol-saturated PBS (0.76 mL) and sample stock solution (0.24 mL) were placed into a 4-mL clear glass vial and incubated on Biosan Thermo Shaker TS-100 at 1000 rpm and 25 °C. For optimization of the assay, the incubation time was varied from 5 h to 48 h. It was found that 24 h was sufficient to reach equilibrium (data not shown). After incubation samples were centrifugated and left for 30 min to ensure complete separation of the phases. The distribution coefficient was determined by quantification of the compounds both in the aqueous and *n*-octanol phases. Then, 100 µL of each phase were transferred to 1.5 mL champagne glass vial and evaporated using a GeneVac EZ-2 PLUS evaporation instrument. Before LC-MS analysis samples were diluted with 200 µL MeCN–0.1% aqueous formic acid (1:1, *v/v*).

LC-MS was performed on Acquity UPLC chromatograph coupled with a MICROMASS QUATTRO micro^TM^ API tandem mass spectrometer. The mass spectrometer was operated in positive electrospray ionisation mode. Parameter settings were as follows: desolvation gas (N_2_) flow was 600 L/h; desolvation gas temperature was 400 °C; capillary voltage, 3.30 kV; ion source temperature was 120 °C. Initial data acquisition was carried out in full-scan mode to select the most abundant ions of the individual compounds. The quantification was achieved in selected ion recording (SIR) mode recording the ions having the highest intensities. Mass-to-charge (*m/z*) values of tested compounds are summarized in Table S1.

Waters Acquity UPLC column BEH C18 (2.1 × 50 mm, 1.7 µm particle size) was applied for analysis. Acetonitrile and 0.1% aqueous formic acid mixtures were used as mobile phase at a flow rate of 0.4 mL/min with the following gradient: 0.0–2.5 min 5–98% MeCN, 2.5–3.5 min 98% MeCN. Injection volume was 2 µL.

Limit of quantitation (LOQ) was determined using S/N ratio for each compound and used for log D_max_ calculations according to the following equation:

(2) $$\log\;\mathrm{Dmax}\;=\;\log\;\left(\frac{\mathrm{theoretical}\;\mathrm{peak}\;\mathrm{area}\;\mathrm{of}\;n-\mathrm{octanol}\;\mathrm{phase}\;\mathrm{at}\;\mathrm{LOQ}\;\mathrm{concentration}}{\mathrm{peak}\;\mathrm{area}\;\mathrm{of}\;\mathrm{buffer}\;\mathrm{base}}\right)$$

Theoretical peak area at LOQ was calculated according to the calibration curve for each compound.

### 3.4. Determination of Minimum Inhibitory Concentration

MIC values of the peptidomimetics were evaluated by using the modified Hancock lab protocol [57] in non-binding polystyrene microtiter plates. Bacterial suspensions of ~5 × 10^5^ CFU/mL in Mueller-Hinton broth (Difco, Sparks, MD, USA) were prepared with Mg^2+^ and Ca^2+^ concentrations of 4 mg/L each. The compounds were dissolved in H_2_O and diluted in 0.01% acetic acid, 0.2% BSA (final concentration). Aliquots (11 µL) of test compounds were then transferred to wells containing bacterial suspensions (100 µL). Low-binding sterile tubes and tips (Axygen, Union City, CA, USA) were used for preparation and transfer of the solutions of the compounds. Following incubation for 20 h of the covered plates at 35 °C (±2 °C) with circular shaking at 180 rpm, the MICs were read. The antibacterial activity of peptidomimetics was tested against *E. coli* ATCC 25922, *K. pneumoniae* ATCC 13883, *P. aeruginosa* PAO1, *A. baumannii* ATCC 19606, *S. aureus* ATCC 29213, *E. faecalis* ATCC 29212, and four MDR strains from the NMI (National Medicines Institute, Warsaw, Poland) collection: *E. coli* NMI 3371/16: carbapenem-resistant, colistin-susceptible (CST-S), and NDM-1-positive (i.e., expressing New Delhi metallo-β-lactamase-1); *E. coli* NMI 3898/15: tigecycline-susceptible (TGC-S, MIC = 0.5), colistin-resistant (CST-R, MIC = 4; mcr1-positive), CMY-2-positive; *P. aeruginosa* NMI 7197/19: colistin-resistant (CST-R, MIC = 16; mcr-negative); *A. baumannii* NMI 3658/17: colistin-resistant (CST-R, MIC > 16; mcr-negative).

### 3.5. Determination of Hemolytic Activity

The lysis of human red blood cells was measured almost as previously described [41], albeit with modifications. In brief, a suspension of freshly drawn human red blood cells (hRBCs) was washed three times with PBS buffer and centrifuged two times for 5 min at 2500 rpm. A two-fold serial dilution of compounds in PBS buffer was prepared. Plates (conical-bottomed 96-well plates) with three replicate wells per dilution, each containing 1% red blood cell suspension, were prepared and dosed with the test compound to achieve a final concentration of 400 μg/mL in a total volume of 100 μL. The plates were incubated (37 °C) for 60 min with mild agitation, and then the cells were pelleted by centrifugation at 1500 rpm for 5 min. The supernatants (50 μL) were then transferred to a fresh 96-well plate, and the concentration of hemoglobin was detected by measuring the OD at 405 nm. The OD of cells incubated with 1% SDS defined 100% hemolysis, while the OD of cells incubated with PBS buffer defined 0% hemolysis. The concentration tested was 400 μg/mL (only average values are stated in Table 5). The percent hemolysis was calculated by using the formula:

(3) $$\mathrm{Hemolysis}\;\left(\%\right)=100\times \left[\frac{A_{\mathrm{sample}}-A_{\mathrm{neg}.\mathrm{control}}}{A_{\mathrm{pos}.\mathrm{control}}-A_{\mathrm{neg}.\mathrm{control}}}\right]$$

### 3.6. Determination of Antiproliferative Activity on HepG2 Cell Line

Effect of compounds on mammalian cell viability was estimated on the HepG2 cell line ATCC HB-8065. In brief, HepG2 cells were seeded into flat-bottomed 96-well plates at a concentration of 5000 cells per well. Subsequently, the cells were incubated for 24 h in a humidified incubator (5% CO_2_, 37 °C). Then, the medium was removed, and the cells were incubated for 48 h in a humidified incubator (5% CO_2_, 37 °C) with peptidomimetics in serial dilutions. Then, an 3-(4,5-dimethylthiazol-2-yl)-2,5-diphenyltetrazolium bromide (MTT) assay was performed as previously described [58]. In brief, after incubation with the test compounds, the culture medium was removed, and then fresh medium with 0.2 mg/mL MTT was added into each well of the plate. The cells were then incubated (3 h, 37 °C, 5% CO_2_), followed by removal of the medium with MTT and addition of 200 μL dimethyl sulfoxide at once to each sample. Absorbance of MTT was measured using the spectrophotometer TECAN Infinite M1000 at 540 nm. The relative viability was calculated by using the formula: OD_treated cells_ × 100/OD_control cells_. The IC_50_ values are calculated by using the program Graph Pad Prism^®^ 5.0. For all compounds, the test range was 10–1280 μg/mL.

## 4. Conclusions

Hydrophobicity of the modified peptidomimetics proved somewhat surprisingly to remain almost constant independently of the degree of sPEG incorporation, since all analogues displaying one to three of these otherwise polarity-inducing residues had peak elution in RP-HPLC within a very narrow range (40.8–42.4% MeCN) including the parent unmodified **1** with intermediate retention (i.e., 41.6% MeCN). Nevertheless, with an increase in the number of oxyethylene moieties incorporated a slight increase in hydrophobity was found.

None of the compounds included in the present study possess activity against *K. pneumoniae* or *E. faecalis*, and against *S. aureus* only analogues displaying a few shorter sPEG moieties exhibited weak activity. Neither length or number of sPEGs incorporated nor their positioning in the sequence appear to influence antibacterial activity against *E. coli,* and none of the sPEG-modified analogues had clearly improved antibacterial activity against *E. coli*. Additionally, for *A. baumannii* and *P. aeruginosa* there appears to be no clear relationship between hydrophobicity and antibacterial activity among these sPEG-modified peptidomimetics. Importantly, the general trend is that antibacterial activity of these peptidomimetics was largely retained against MDR *E. coli* strains, including the colistin-resistant (mcr1-positive) *E. coli* isolate. Similarly, colistin resistance in *A. baumannii* did not appear to influence the susceptibility of this strain toward the peptidomimetics.

The hemolytic properties of the entire series of peptidomimetics were relatively modest (i.e., within the range 2.4–9.4% at 400 µg/mL) indicating a retained acceptable cell selectivity even at 50- to 100-fold higher concentration than the typical MICs against *E. coli* or *A. baumannii*. While the number of sPEG residues did not appear to be closely correlated with hemolytic activity, a trend toward lowered hemolytic activity was observed for analogues displaying shorter sPEG moieties. In contrast, HepG2 cell viability was affected the least by analogues, incorporating two longer sPEG residues (i.e., sPEG3 and sPEG4), which had IC_50_ values of ~1280 µg/mL (the highest concentration tested).

Based on the present findings the most favorable modifications of peptide/β-peptoid hybrid oligomers appear to involve incorporation of one or two extended sPEG moieties, providing increased cell selectivity while retaining potent antibacterial activity (e.g., **10** with a MIC of 1.3 µM against *A. baumannii* as well as **16** and **18** with MICs of 0.7–1.4 µM and 1.2–2.4 µM, respectively, against *E. coli*).

Future studies of this novel class of peptidomimetics may comprise further characterization of the physicochemical properties (e.g., hydrodynamic radius and critical micelle concentration as well as potential for other types of aggregation), validation of proteolytic stability, testing against a wide range of clinical isolates and additional mammalian cell lines as well as efficacy in animal infection models. In addition, it may be envisioned that modification with sPEGs may prove to constitute a useful tool for optimization of other types of peptidomimetics.

## Figures and Tables

**Table 2:** Type of column and data supplied by manufacturers.

Supplier and Type of Column	Stationary Phase	Dimensions	Particle Size [µm]	Pore Size [Å]	Reference
Phenomenex, Luna	C18(2)	150 × 4.6 mm	3.0	100	[49]
Phenomenex, Luna	Omega Polar C18	150 × 4.6 mm	3.0	100	[50]
Phenomenex, Aeris	Peptide XB-C18	150 × 4.6 mm	3.6	100	[51]
Sigma-Aldrich, Supelco Discovery BIO Wide pore	C18	150 × 4.6 mm	3.0	300	[52]

**Table 3:** Minimum inhibitory concentrations (MIC values) for oligomer **1**–**18**.

Subgroup	Cmpd	Sequence ^1^	% MeCN ^2^	MIC [µg/mL]					
				Gram-Negative ^3^				Gram-Positive ^4^	
				EC	KP	PA	AB	SA	EF
	**1**	H-[X]_6_-NH_2_	41.6	4–8	64–>64	32–64	8–16	16	>64
I	**2**	H-sPEG2-[X]_6_-NH_2_	41.4	8	>64	32	4	32–64	>64
	**3**	H-sPEG4-[X]_6_-NH_2_	41.9	8	>64	32–64	4–8	64–>64	>64
II	**4**	H-sPEG2-[X]_6_-sPEG2-NH_2_	41.3	8–16	>64	16	4	>64	>64
	**5**	H-sPEG4-[X]_6_-sPEG4-NH_2_	42.2	16	>64	>64	8–16	>64	>64
III	**6**	H-[X]_3_-sPEG1-[X]_3_-NH_2_	41.2	4	64–>64	32	4–8	32–64	64–>64
	**7**	H-[X]_3_-sPEG2-[X]_3_-NH_2_	41.4	4	>64	16–32	4	64–>64	64–>64
	**8**	H-[X]_3_-sPEG3-[X]_3_-NH_2_	41.7	4	>64	32	4–8	64–>64	>64
	**9**	H-[X]_3_-sPEG4-[X]_3_-NH_2_	41.8	4	>64	32	4–8	>64	>64
	**10**	H-[X]_3_-sPEG6-[X]_3_-NH_2_	42.3	4–8	64	16	4	>64	>64
IV	**11**	H-sPEG1-[X]_3_-sPEG1-[X]_3_-sPEG1-NH_2_	40.8	8	>64	32	4–8	>64	>64
	**12**	H-sPEG2-[X]_3_-sPEG2-[X]_3_-sPEG2-NH_2_	41.3	8–16	>64	16–64	8–16	>64	>64
	**13**	H-sPEG3-[X]_3_-sPEG3-[X]_3_-sPEG3-NH_2_	41.9	8–16	>64	>64	8–16	>64	>64
	**14**	H-sPEG4-[X]_3_-sPEG4-[X]_3_-sPEG4-NH_2_	42.4	16	>64	>64	16	>64	>64
V	**15**	H-[X]_2_-sPEG1-[X]_2_-sPEG1-[X]_2_-NH_2_	41.1	4	64–>64	32–64	8	>64	>64
	**16**	H-[X]_2_-sPEG2-[X]_2_-sPEG2-[X]_2_-NH_2_	41.5	2–4	64	32–64	8	32–64	>64
	**17**	H-[X]_2_-sPEG3-[X]_2_-sPEG3-[X]_2_-NH_2_	41.8	8	>64	>64	16–32	>64	>64
	**18**	H-[X]_2_-sPEG4-[X]_2_-sPEG4-[X]_2_-NH_2_	42.1	4–8	64	>64	8–16	>64	>64

**^1^** X = Lys-βNPhe(4-F). **^2^** Experimental relative hydrophobicity measured as % MeCN at peak elution. RP-HPLC gradient: 0–60% B during 15 min. **^3^** EC: *E. coli* ATCC 25922; KP: *K. pneumoniae* ATCC 13883; PA: *P. aeruginosa* PAO1; AB: *A. baumannii* ATCC 19606. **^4^** SA: *S. aureus* ATCC 29213; EF: *E. faecalis* ATCC 29212.

**Table 4:** Minimum inhibitory concentrations (MIC values) against bacteria possessing AMR.

Subgroup	Cmpd	*E. coli* ^1^			*P. aeruginosa* ^2^		*A. baumannii* ^3^	
		WT	NMI 3898/15	NMI 3371/16	WT	NMI 7197/19	WT	NMI 3658/17
	**1**	4–8	8	8	32–64	>64	8–16	4
I	**2**	8	16	8	32	>64	4	4
	**3**	8	16	8	32–64	>64	4–8	4–8
II	**4**	8–16	16–32	8–16	16	>64	4	4–8
	**5**	16	32	8–16	>64	>64	8–16	16
III	**6**	4	8	4	32	>64	4–8	4
	**7**	4	8	4	16–32	>64	4	4
	**8**	4	8	4	32	>64	4–8	4
	**9**	4	16	4–8	32	>64	4–8	4–8
	**10**	4–8	8–32	4–16	16	>64	4	4
IV	**11**	8	16	8	32	>64	4–8	8
	**12**	8–16	16	8	16–64	>64	8–16	16
	**13**	8–16	16	8	>64	>64	8–16	16
	**14**	16	32	16	>64	>64	16	32
V	**15**	4	8	4	32–64	>64	8	4
	**16**	2–4	8	4	32–64	>64	8	4
	**17**	8	32	8–16	>64	>64	16–32	16
	**18**	4–8	16	8–16	>64	>64	8–16	16

NMI: National Medicine Institute, Warsaw, Poland. **^1^** WT: *E. coli* ATCC 25922; *E. coli* NMI 3898/15: tigecycline-susceptible (TGC-S, MIC = 0.5), colistin-resistant (CST-R, MIC = 4; mcr1-positive), CMY-2-positive; *E. coli* NMI 3371/16: carbapenem-resistant, colistin-susceptible (CST-S), and NDM-1-positive (i.e., expressing New Delhi metallo-β-lactamase-1) [56]. **^2^** WT: *P. aeruginosa* PAO1; *P. aeruginosa* NMI 7197/19: colistin-resistant (CST-R, MIC = 16; mcr-negative). **^3^** WT: *A. baumannii* ATCC 19606; *A. baumannii* NMI 3658/17: colistin-resistant (CST-R, MIC >16; mcr-negative).

**Table 5:** Hemolytic activity for all compounds and effect on HepG2 cell viability for compounds **1**–**18**.

Subgroup	Cmpd	Sequence ^1^	% MeCN ^2^	Hemolysis ^3^	HepG2 IC_50_ [µg/mL] ^4^	SI ^5^
	**1**	H-[X]_6_-NH_2_	41.6	6.6	464 (0%)	58–116
I	**2**	H-sPEG2-[X]_6_-NH_2_	41.4	2.4	n.d.	n.d.
	**3**	H-sPEG4-[X]_6_-NH_2_	41.9	8.1	n.d.	n.d.
II	**4**	H-sPEG2-[X]_6_-PEG2-NH_2_	41.3	5.4	n.d.	n.d.
	**5**	H-sPEG4-[X]_6_-PEG4-NH_2_	42.2	6.4	n.d.	n.d.
III	**6**	H-[X]_3_-sPEG1-[X]_3_-NH_2_	41.2	7.7	761 (6%)	190
	**7**	H-[X]_3_-sPEG2-[X]_3_-NH_2_	41.4	6.1	619 (10%)	155
	**8**	H-[X]_3_-sPEG3-[X]_3_-NH_2_	41.7	8.9	601 (10%)	150
	**9**	H-[X]_3_-sPEG4-[X]_3_-NH_2_	41.8	7.1	693 (9%)	173
	**10**	H-[X]_3_-sPEG6-[X]_3_-NH_2_	42.3	6.3	800 (27%)	100–200
IV	**11**	H-sPEG1-[X]_3_-sPEG1-[X]_3_-sPEG1-NH_2_	40.8	4.6	n.d.	n.d.
	**12**	H-sPEG2-[X]_3_-sPEG2-[X]_3_-sPEG2-NH_2_	41.3	8.1	332 (34%)	21–42
	**13**	H-sPEG3-[X]_3_-sPEG3-[X]_3_-sPEG3-NH_2_	41.9	7.8	n.d.	n.d.
	**14**	H-sPEG4-[X]_3_-sPEG4-[X]_3_-sPEG4-NH_2_	42.4	9.0	450 (44%)	28
V	**15**	H-[X]_2_-sPEG1-[X]_2_-sPEG1-[X]_2_-NH_2_	41.1	4.8	568 (26%)	142
	**16**	H-[X]_2_-sPEG2-[X]_2_-sPEG2-[X]_2_-NH_2_	41.5	5.7	687 (27%)	172–344
	**17**	H-[X]_2_-sPEG3-[X]_2_-sPEG3-[X]_2_-NH_2_	41.8	9.4	~1280 (35%)	160
	**18**	H-[X]_2_-sPEG4-[X]_2_-sPEG4-[X]_2_-NH_2_	42.1	8.3	~1280 (41%)	160–320

**^1^** X = Lys-βNPhe(4-F). **^2^** Experimental relative hydrophobicity measured as % MeCN at peak elution. RP-HPLC gradient: 0–60% B during 15 min. **^3^** Percentage hemolysis in human red blood cells (hRBCs) at 400 µg/mL. **^4^** Toxicity against HepG2 cells is given as the average IC_50_ value for inhibition of growth of HepG2 cells (assay was performed in two biological replicates each with three technical replicates). The highest concentration tested was 1280 µg/mL, HepG2 cell viability at this concentration is given in parenthesis. n.d. = not determined. **^5^** Selectivity Index (SI) was calculated as the ratio between IC_50_ for effect on HepG2 cell viability and MIC against *E. coli*. n.d. = not determined.

## Data Availability

The data presented in this study are available in the present article and Supplementary Materials.

## Supplementary Materials

The following are available online at https://www.mdpi.com/article/10.3390/ijms22137041/s1, Table S1: Log D_max_ values and selected ion recording parameters of compounds, Table S2: Calculated log D values of compounds, Characterization of peptidomimetics: HRMS spectra and HPLC chromatograms, HepG2 IC_50_-curves.
