# Supplementary material for: Peptide/β-Peptoid Hybrids with Ultrashort PEG-Like Moieties: Effects on Hydrophobicity, Antibacterial Activity and Hemolytic Properties
Source: Int J Mol Sci. 2021 Jun 30;22(13):7041. doi: 10.3390/ijms22137041 (PMC8268887; doi:10.3390/ijms22137041)

International Journal of *Molecular Sciences*

MDPI

*Supplementary Material*

# Peptide/β-Peptoid Hybrids with Ultrashort PEG-Like Moieties: Effects on Hydrophobicity, Antibacterial Activity and Hemolytic Properties

**Nicki Frederiksen [1], Stavroula Louka [1], Chirag Mudaliar [1], Ilona Domraceva [2], Agrita Kreicberga [2], Osvalds Pugovics [2], Dorota Żabicka [3], Magdalena Tomczak [3], Weronika Wygoda [3], Fredrik Björkling [1] and Henrik Franzyk [1,*]**

[1] Center for Peptide-Based Antibiotics, Department of Drug Design and Pharmacology, Faculty of Health and Medical Sciences, University of Copenhagen, DK-2100 Copenhagen, Denmark; nicki.frederiksen@sund.ku.dk (N.F.); stavroulaloukad@gmail.com (S.L.); chiragmudaliar@gmail.com (C.M.); fb@sund.ku.dk (F.B.)
[2] Latvian Institute of Organic Synthesis, Aizkraukles 21, 1006 Riga, Latvia; ilona@farm.osi.lv (I.D.); agrita.kreicberga@osi.lv (A.K.); osvalds@osi.lv (O.P.)
[3] Department of Epidemiology and Clinical Microbiology, National Medicines Institute, ul. Chełmska 30/34, 00-725 Warsaw, Poland; d.zabicka@nil.gov.pl (D.Ż.); mtomczak@cls.edu.pl (M.T.); w.wygoda@nil.gov.pl (W.W.)
* Correspondence: henrik.franzyk@sund.ku.dk

## Contents

2 ........ Estimation of log D values

3 ........ Calculation of log D values

4 ........ Characterization of peptidomimetics

22 ........ HepG2 $IC_{50}$ curves

## Estimation of log D values

**Table S1.** Log $D_{max}$ values and selected ion recording parameters of compounds.

| Compound | log $D_{max}$ | m/z | | |
|---|---|---|---|---|
| | | z = 6 | z = 5 | z = 4 |
| **1** | <−2.662 | 373.3 | 466.3 | 621.4 |
| **7** | <−3.650 | 402.3 | 502.6 | 669.8 |
| **9** | <−3.627 | 352.5 | 422.7 | 527.2 |
| **10** | <−2.994 | 367.1 | 440.4 | 550.2 |
| **11** | <−2.876 | 361.8 | 433.9 | 542.1 |
| **12** | <−3.147 | 383.8 | 460.4 | 575.2 |
| **13** | <−3.911 | 405.8 | 486.8 | 608.3 |
| **14** | <−2.883 | 434.9 | 521.7 | 651.9 |
| **15** | <−3.180 | 345.0 | 413.7 | 516.9 |
| **16** | <−3.922 | 359.5 | 431.2 | 538.8 |

## Calculation of log D values

**Table S2**. Calculated log D values of compounds.

| Subgroup | Cmpd | Sequence [1] | %MeCN [2] | *cLogD*$_{7.4}$ [3] |
|---|---|---|---|---|
| I | **1** | H-[X]$_6$-NH$_2$ | 41.6 | −14.22 |
| | **2** | H-sPEG2-[X]$_6$-NH$_2$ | 41.4 | −16.22 |
| | **3** | H-sPEG4-[X]$_6$-NH$_2$ | 41.9 | −16.08 |
| II | **4** | H-sPEG2-[X]$_6$-sPEG2-NH$_2$ | 41.3 | −17.42 |
| | **5** | H-sPEG4-[X]$_6$-sPEG4-NH$_2$ | 42.2 | −17.13 |
| III | **6** | H-[X]$_3$-sPEG1-[X]$_3$-NH$_2$ | 41.2 | −15.37 |
| | **7** | H-[X]$_3$-sPEG2-[X]$_3$-NH$_2$ | 41.4 | −15.41 |
| | **8** | H-[X]$_3$-sPEG3-[X]$_3$-NH$_2$ | 41.7 | −15.46 |
| | **9** | H-[X]$_3$-sPEG4-[X]$_3$-NH$_2$ | 41.8 | −15.27 |
| | **10** | H-[X]$_3$-sPEG6-[X]$_3$-NH$_2$ | 42.3 | −15.37 |
| IV | **11** | H-sPEG1-[X]$_3$-sPEG1-[X]$_3$-sPEG1-NH$_2$ | 40.8 | −18.20 |
| | **12** | H-sPEG2-[X]$_3$-sPEG2-[X]$_3$-sPEG2-NH$_2$ | 41.3 | −18.62 |
| | **13** | H-sPEG3-[X]$_3$-sPEG3-[X]$_3$-sPEG3-NH$_2$ | 41.9 | −18.76 |
| | **14** | H-sPEG4-[X]$_3$-sPEG4-[X]$_3$-sPEG4-NH$_2$ | 42.4 | −18.19 |
| V | **15** | H-[X]$_2$-sPEG1-[X]$_2$-sPEG1-[X]$_2$-NH$_2$ | 41.1 | −16.52 |
| | **16** | H-[X]$_2$-sPEG2-[X]$_2$-sPEG2-[X]$_2$-NH$_2$ | 41.5 | −16.61 |
| | **17** | H-[X]$_2$-sPEG3-[X]$_2$-sPEG3-[X]$_2$-NH$_2$ | 41.8 | −16.71 |
| | **18** | H-[X]$_2$-sPEG4-[X]$_2$-sPEG4-[X]$_2$-NH$_2$ | 42.1 | −16.33 |

[1] X = Lys-βNPhe(4-F). [2] Experimental relative hydrophobicity measured as % MeCN at peak elution. RP-HPLC gradient: 0 → 60% B during 15 min. For column specifications, see **Error! Reference source not found.**. [3] *cLogD*$_{7.4}$ = calculated distribution coefficient at pH 7.4.

## Characterization of peptidomimetics

## Compound 1

**HRMS:** calculated for $[M+H]^{1+}$ 1862.05481, found 1862.05565; ΔM = 0.5 ppm.

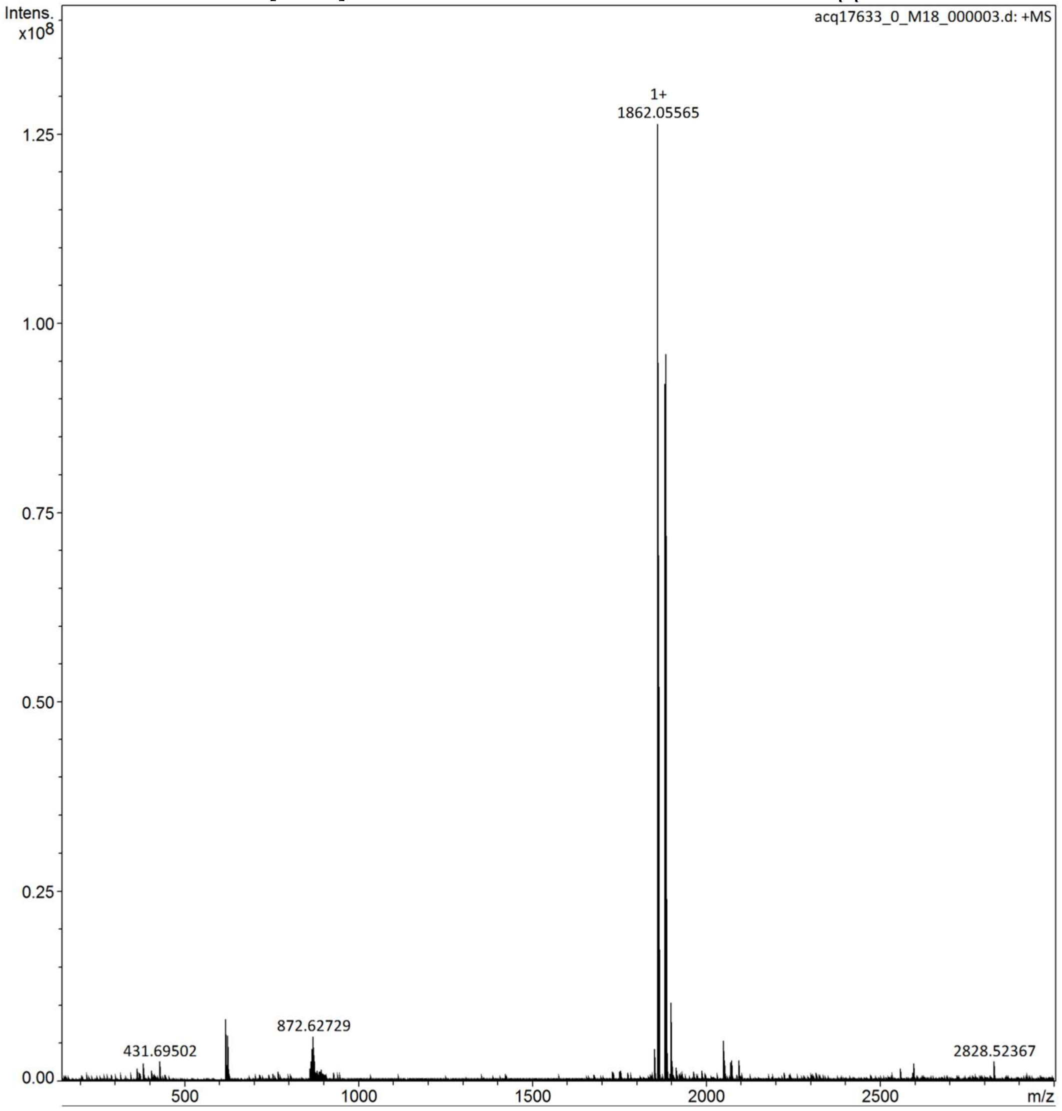


**HPLC:** Charge: +7. $t_R$ = 6.534 min, purity 98.131%. Gradient: 0-60% B during 10 min. B = 95% MeCN + 0.1% TFA.

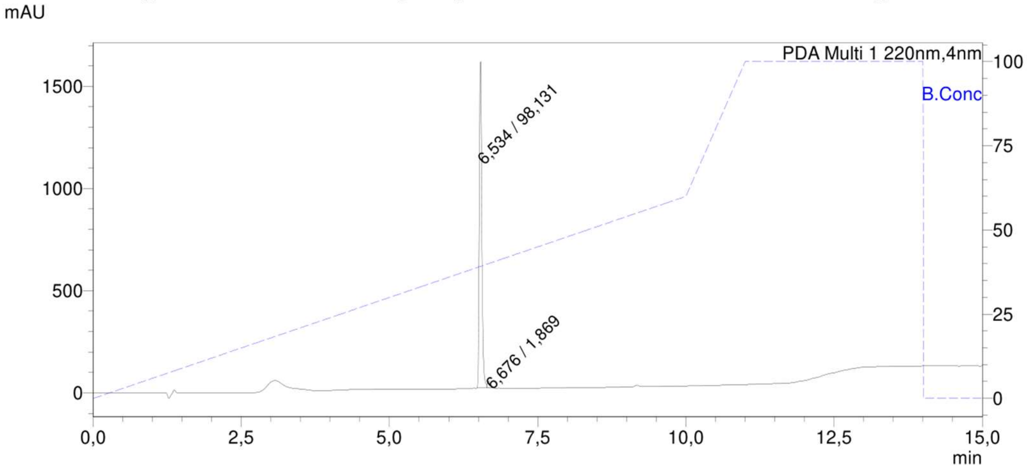

## Compound 2

**HRMS:** calculated for $[M+H]^{1+}$ 2007.12870, found 2007.12894; ΔM = 0.1 ppm.

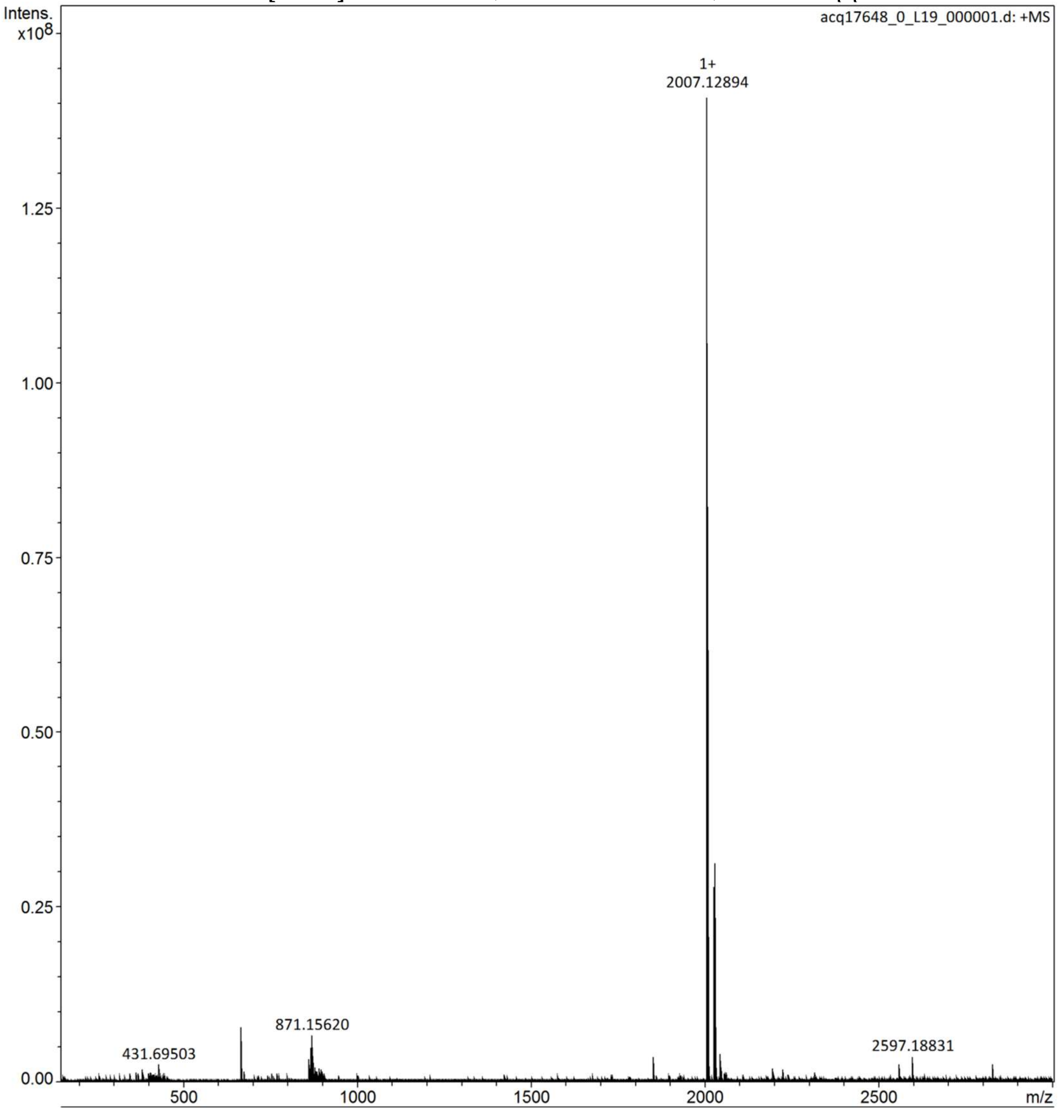


**HPLC:** Charge: +7. $t_R$ = 6.524 min, purity 99.102%. Gradient: 0-60% B during 10 min. B = 95% MeCN + 0.1% TFA.

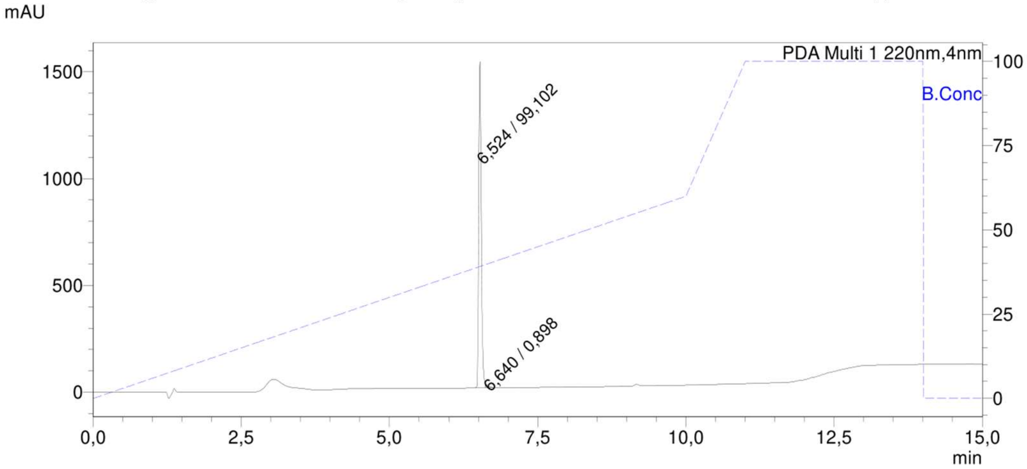

**Compound 3**

**HRMS:** calculated for $[M+H]^{1+}$ 2109.19678, found 2109.19714Y; ΔM = 0.2 ppm.

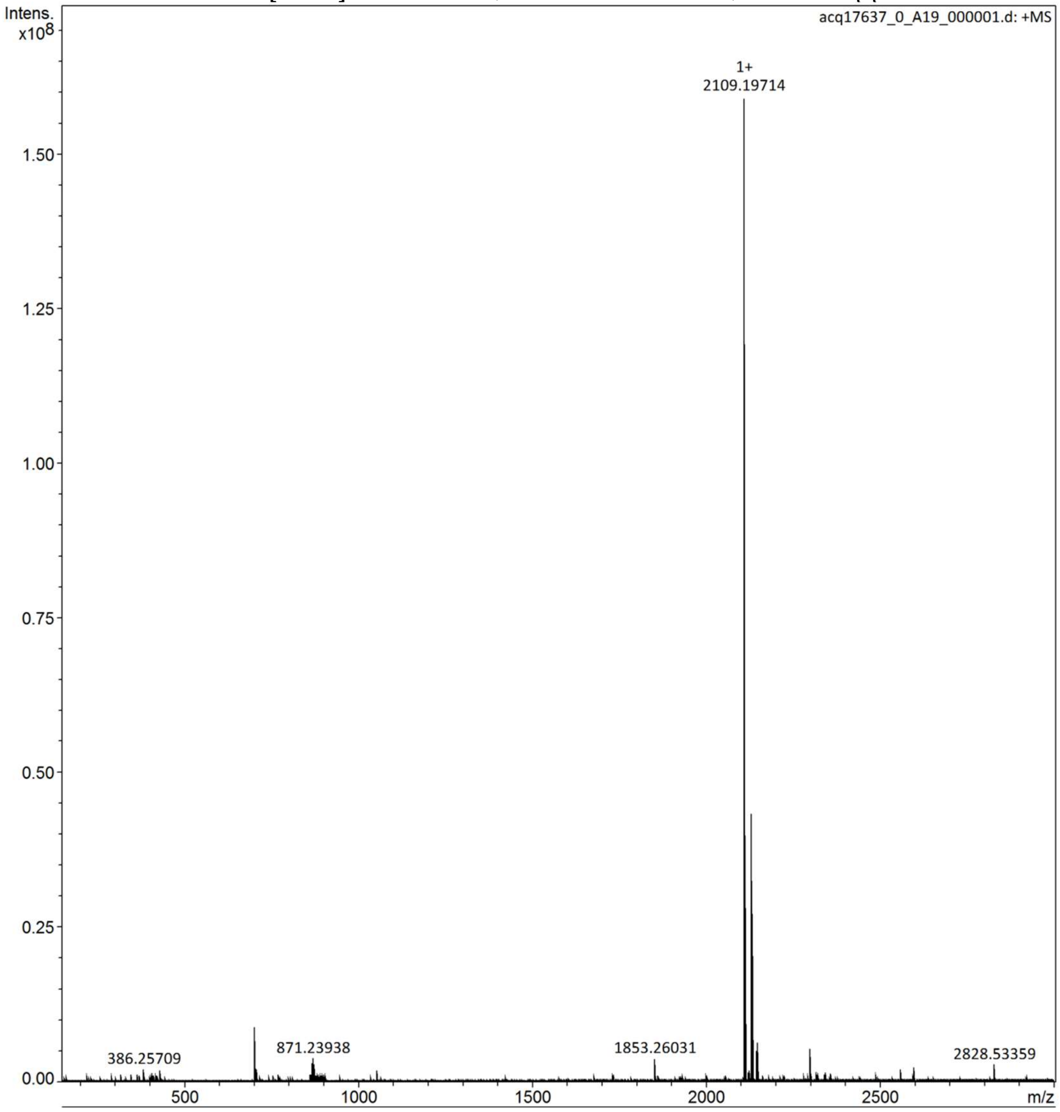


**HPLC:** Charge: +7. $t_R$ = 6.579 min, purity 99.368%. Gradient: 0-60% B during 10 min. B = 95% MeCN + 0.1% TFA.

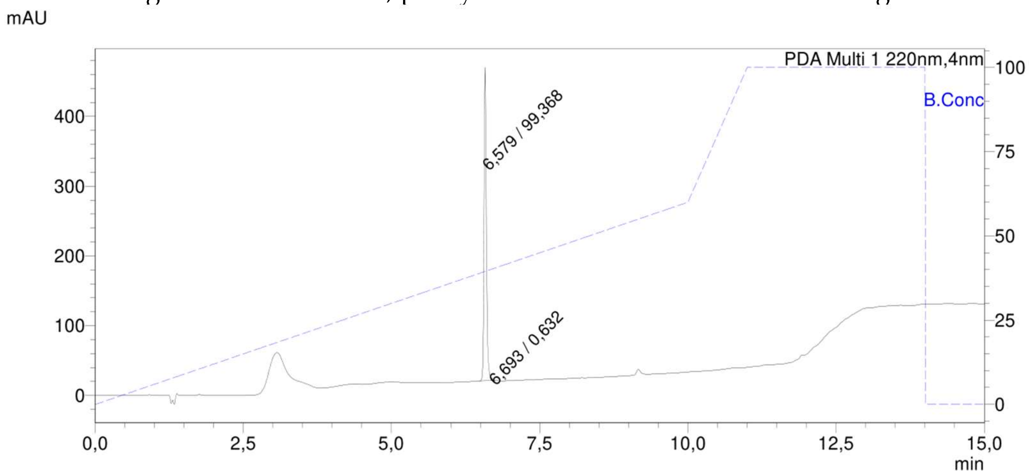

## Compound 4

**HRMS:** calculated for [M+H]$^{1+}$ 2152.20260, found 2152.20369; ΔM = 0.5 ppm.

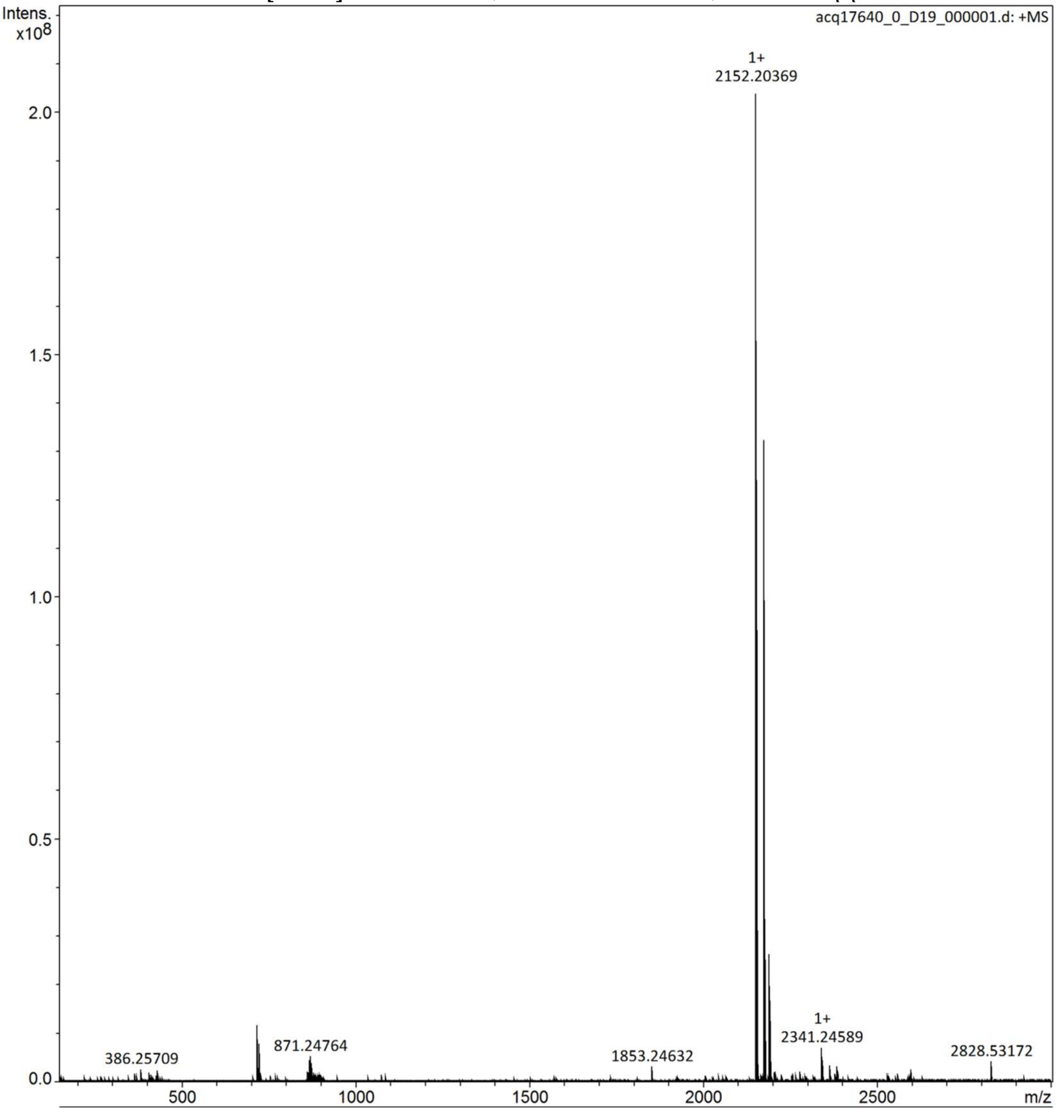


**HPLC:** Charge: +7. $t_R$ = 6.514 min, purity 99.670%. Gradient: 0-60% B during 10 min. B = 95% MeCN + 0.1% TFA.

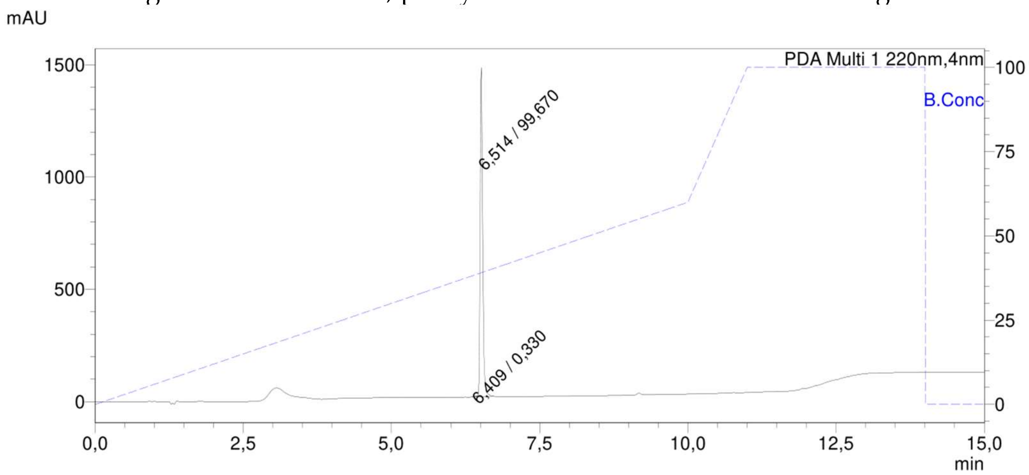

**Compound 5**

**HRMS:** calculated for [M+H]$^{1+}$ 2356.33876, found 2356.33774; ΔM = 0.4 ppm.

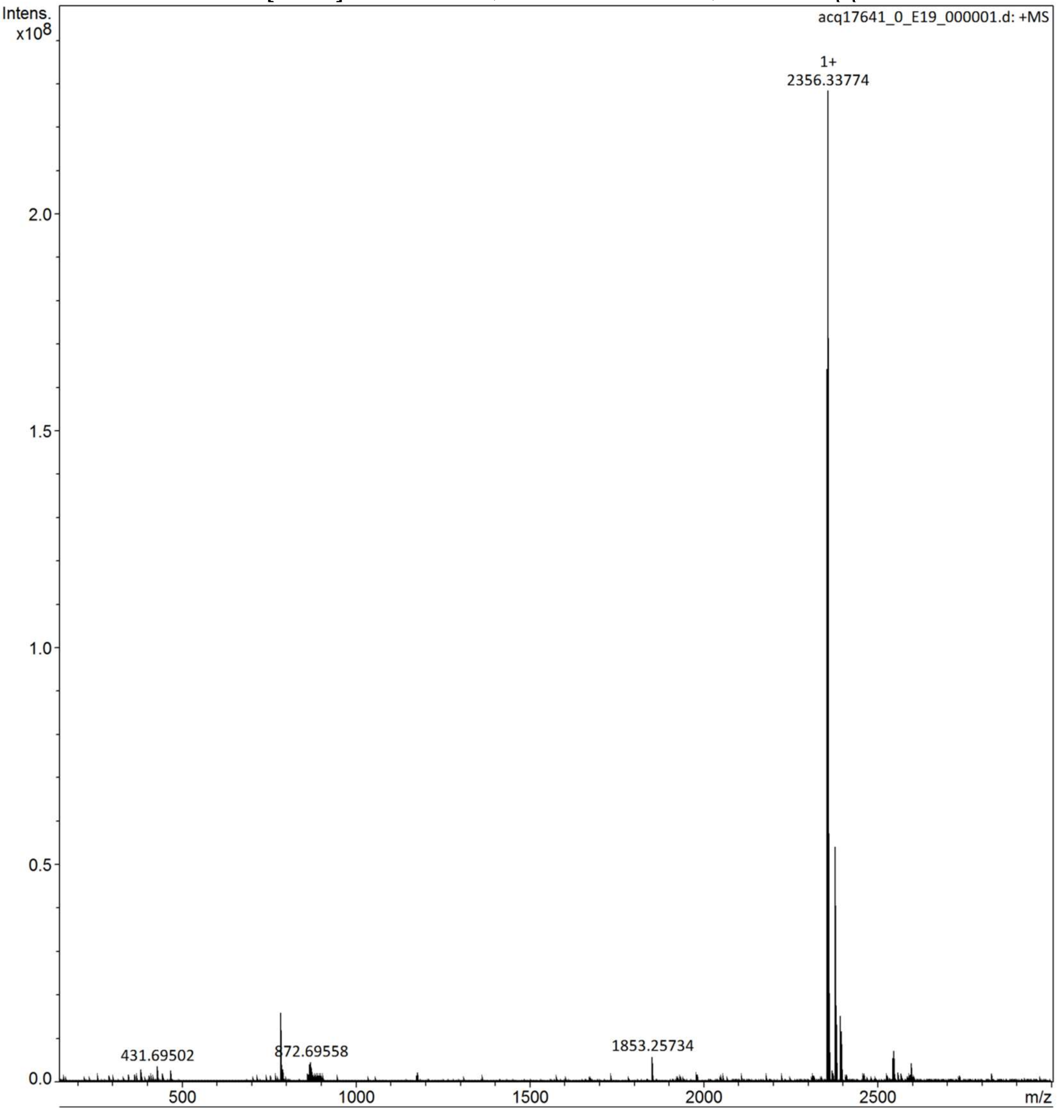


**HPLC:** Charge: +7. $t_R$ = 6.638 min, purity 98.112%. Gradient: 0-60% B during 10 min. B = 95% MeCN + 0.1% TFA.

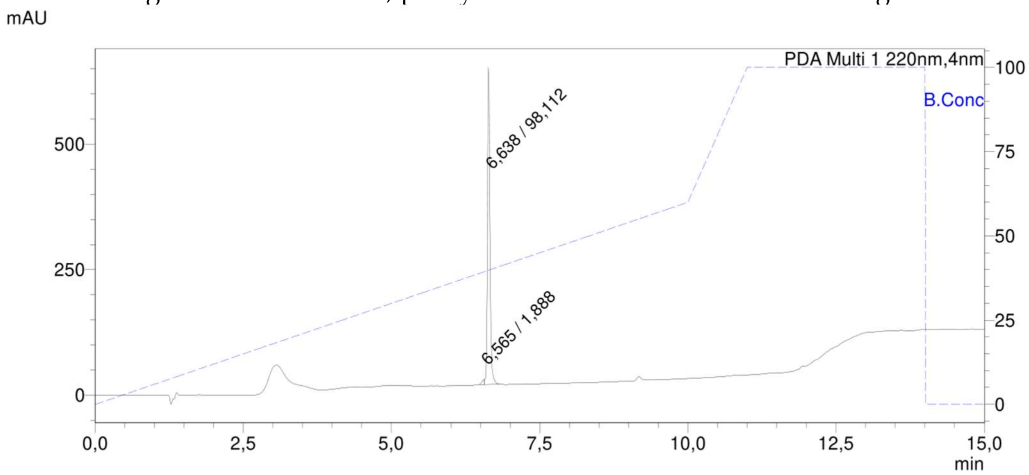

## Compound 6

**HRMS:** calculated for $[M+H]^{1+}$ 1963.10249, found 1963.10271; ΔM = 0.1 ppm.

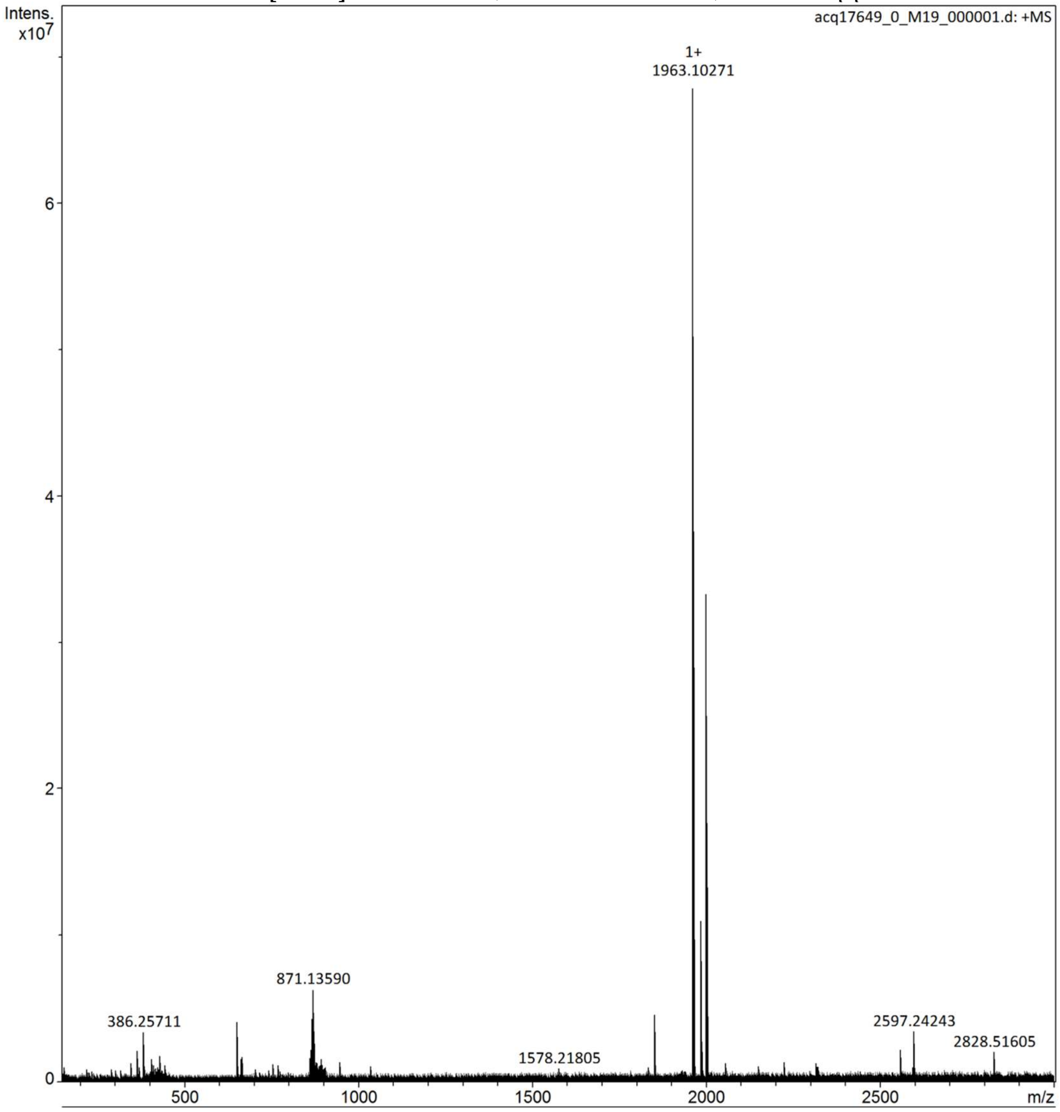


**HPLC:** Charge: +7. $t_R$ = 6.505 min, purity 98.327%. Gradient: 0-60% B during 10 min. B = 95% MeCN + 0.1% TFA.

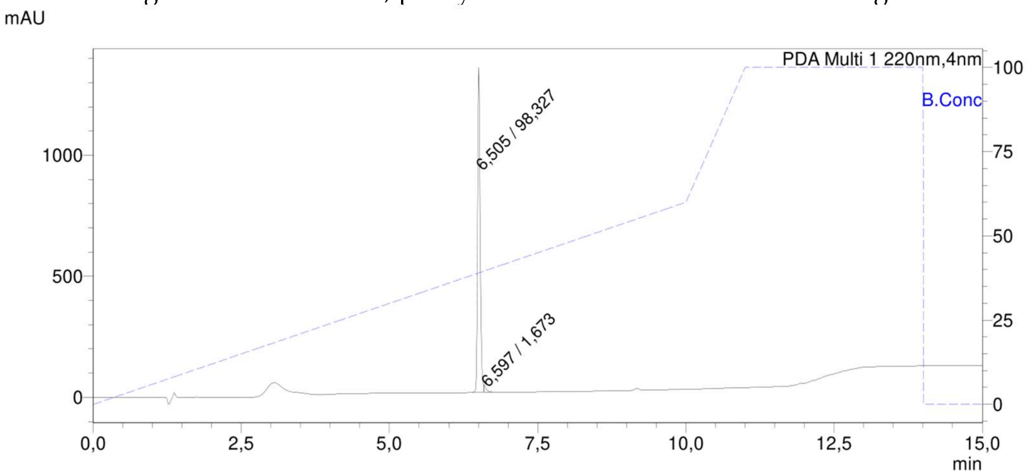

**Compound 7**

**HRMS:** calculated for $[M+H]^{1+}$ 2007.12870, found 2007.12950; ΔM = 0.4 ppm.

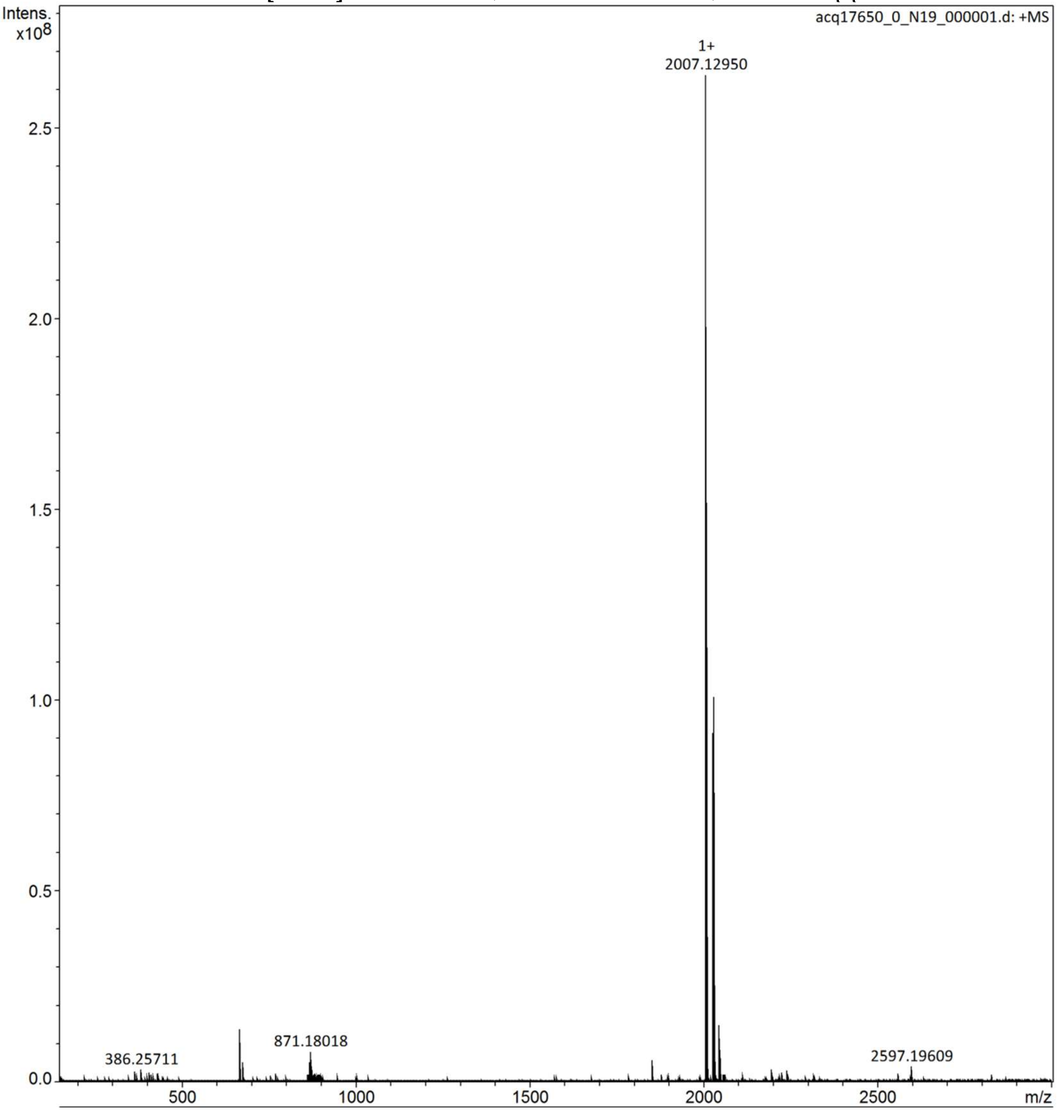


**HPLC:** Charge: +7. $t_R$ = 6.528 min, purity 98.548%. Gradient: 0-60% B during 10 min. B = 95% MeCN + 0.1% TFA.

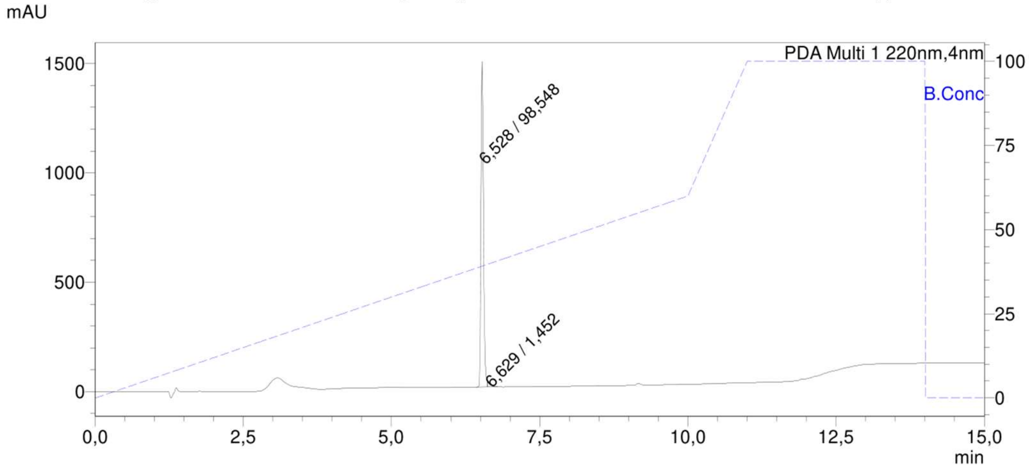

## Compound 8

**HRMS:** calculated for $[M+H]^{1+}$ 2051.15492, found 2051.15671; ΔM = 0.9 ppm.

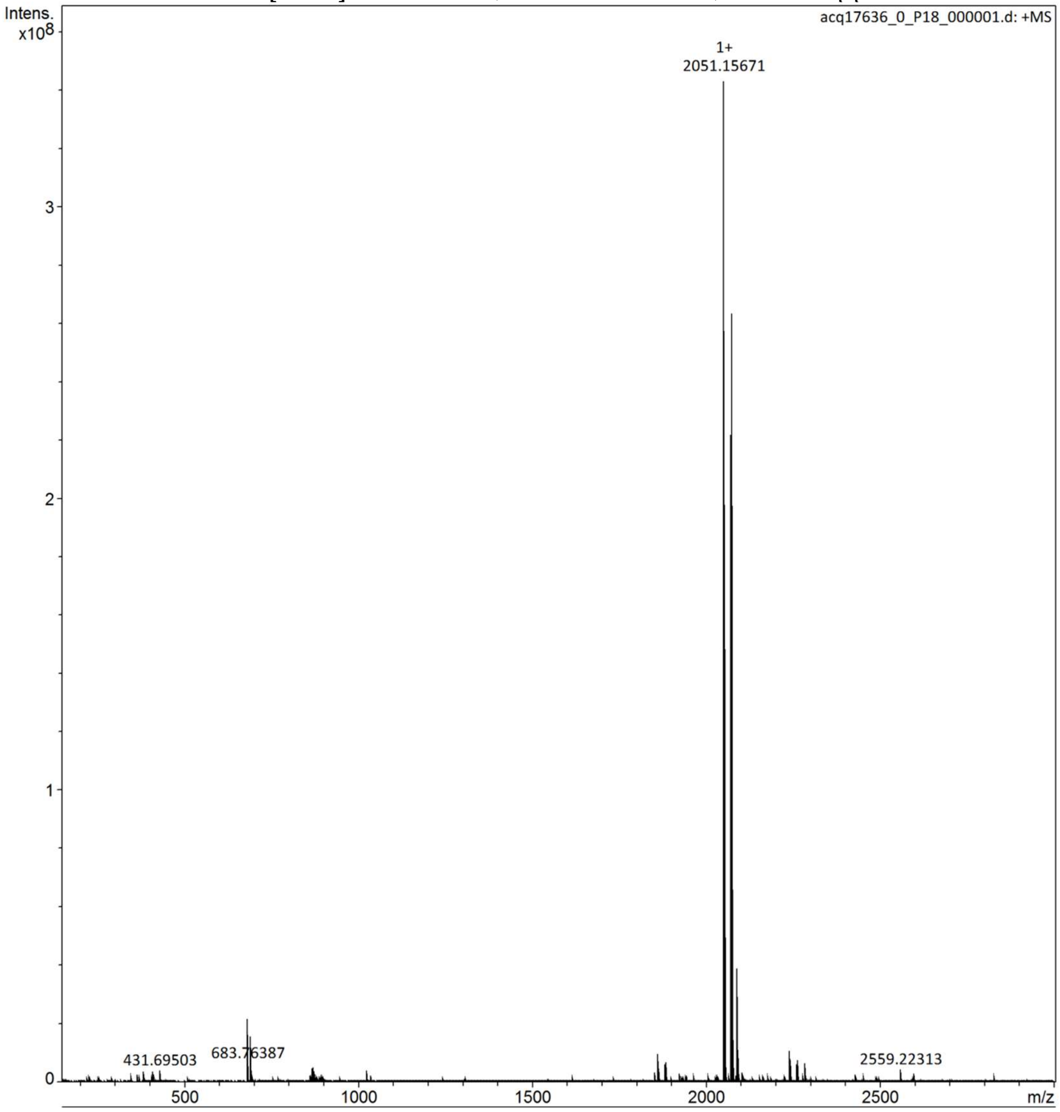


**HPLC:** Charge: +7. $t_R$ = 6.569 min, purity 99.294%. Gradient: 0-60% B during 10 min. B = 95% MeCN + 0.1% TFA.

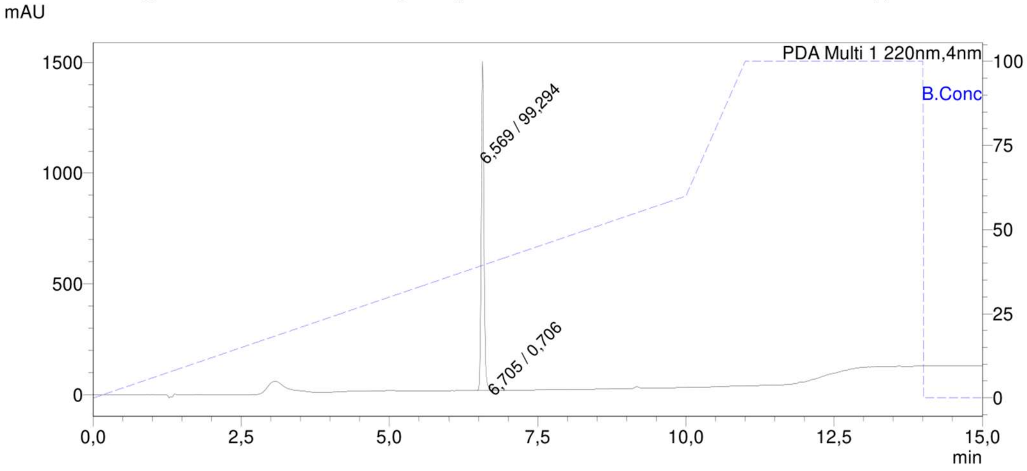

**Compound 9**

**HRMS:** calculated for $[M+H]^{1+}$ 2109.19678, found 2109.19733; ΔM = 0.3 ppm.

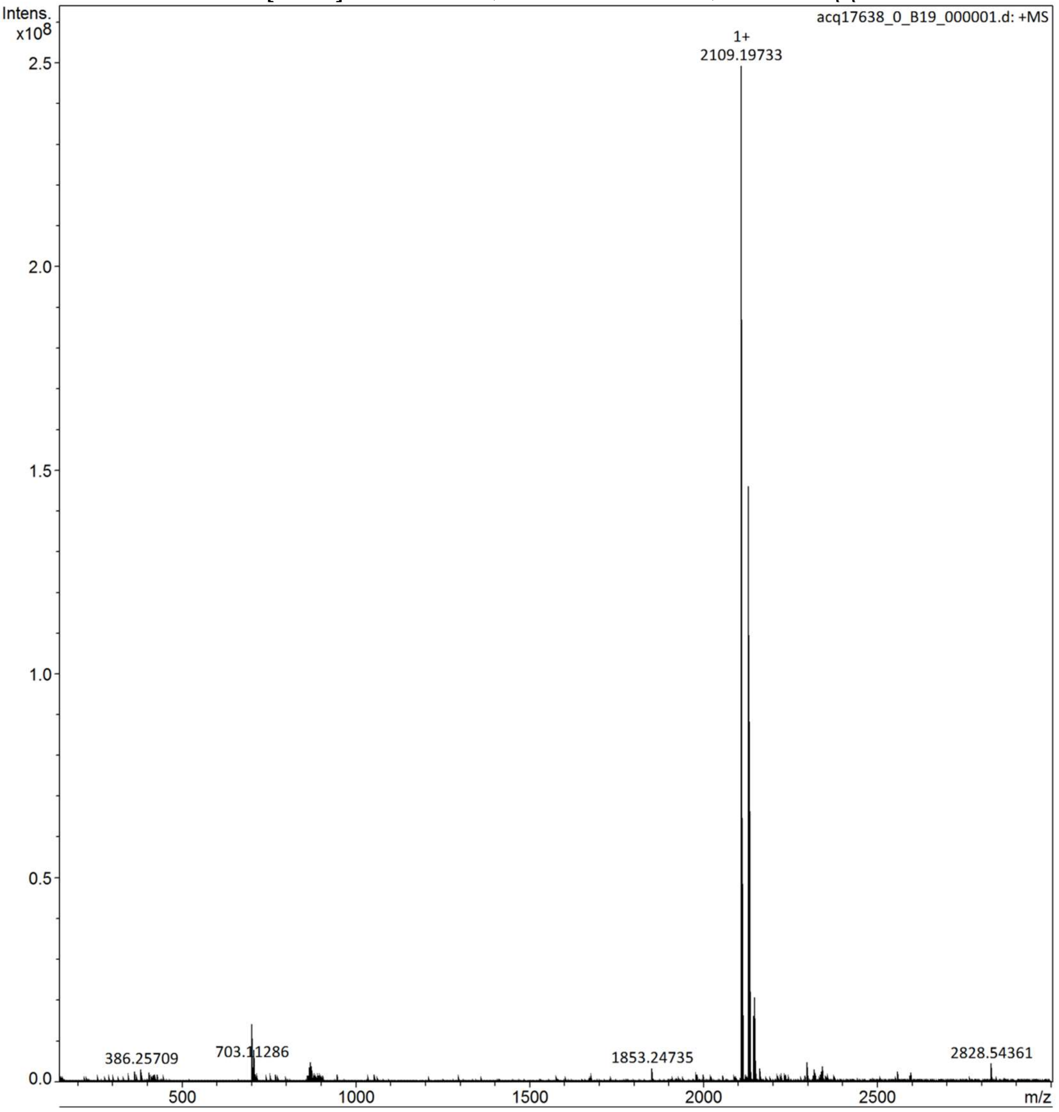


**HPLC:** Charge: +7. $t_R$ = 6.588 min, purity 99.198%. Gradient: 0-60% B during 10 min. B = 95% MeCN + 0.1% TFA.

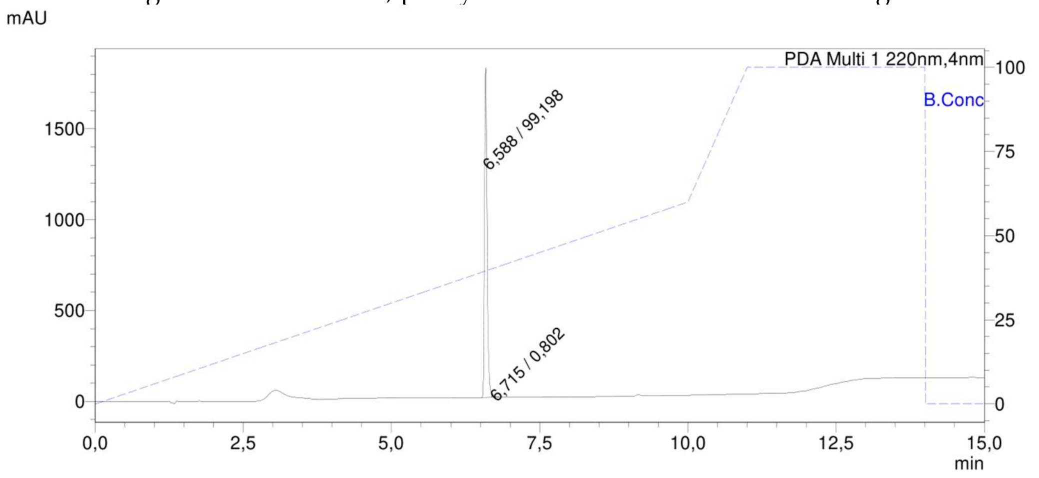

**Compound 10**

**HRMS:** calculated for $[M+H]^{1+}$ 2197.24921, found 2197.25033; ΔM = 0.5 ppm.

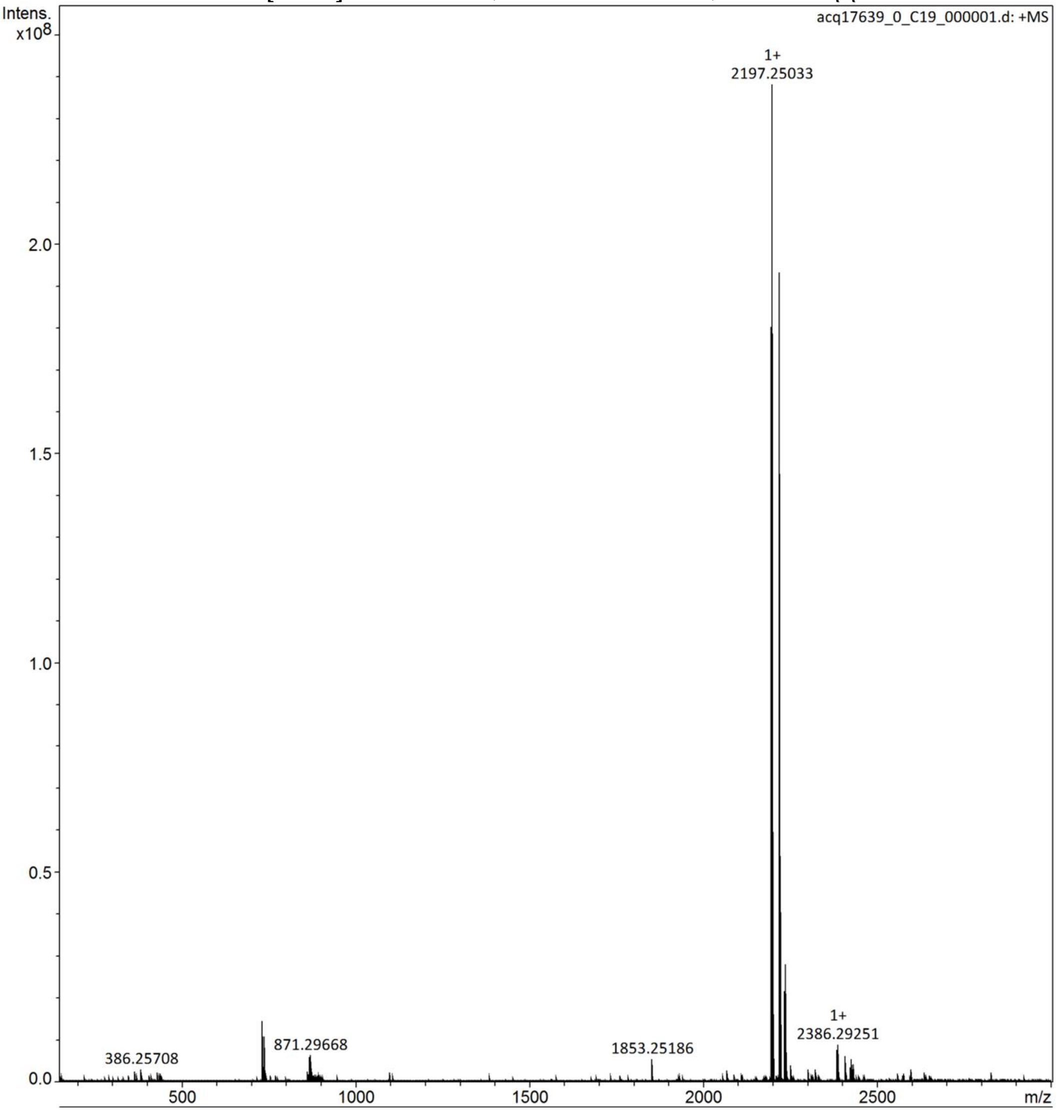


**HPLC:** Charge: +7. $t_R$ = 6.664 min, purity 99.251%. Gradient: 0-60% B during 10 min. B = 95% MeCN + 0.1% TFA.

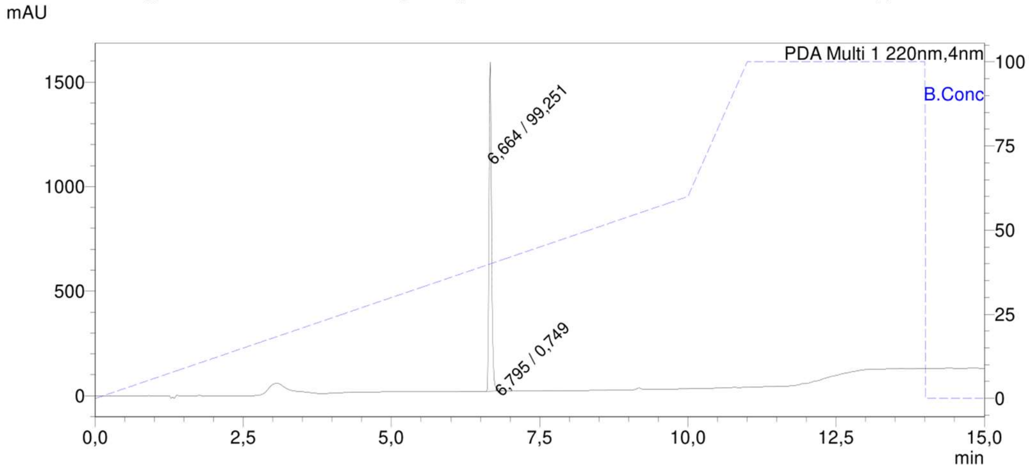

**Compound 11**

**HRMS:** calculated for $[M+H]^{1+}$ 2165.19785, found 2165.19755; ΔM = 0.1 ppm.

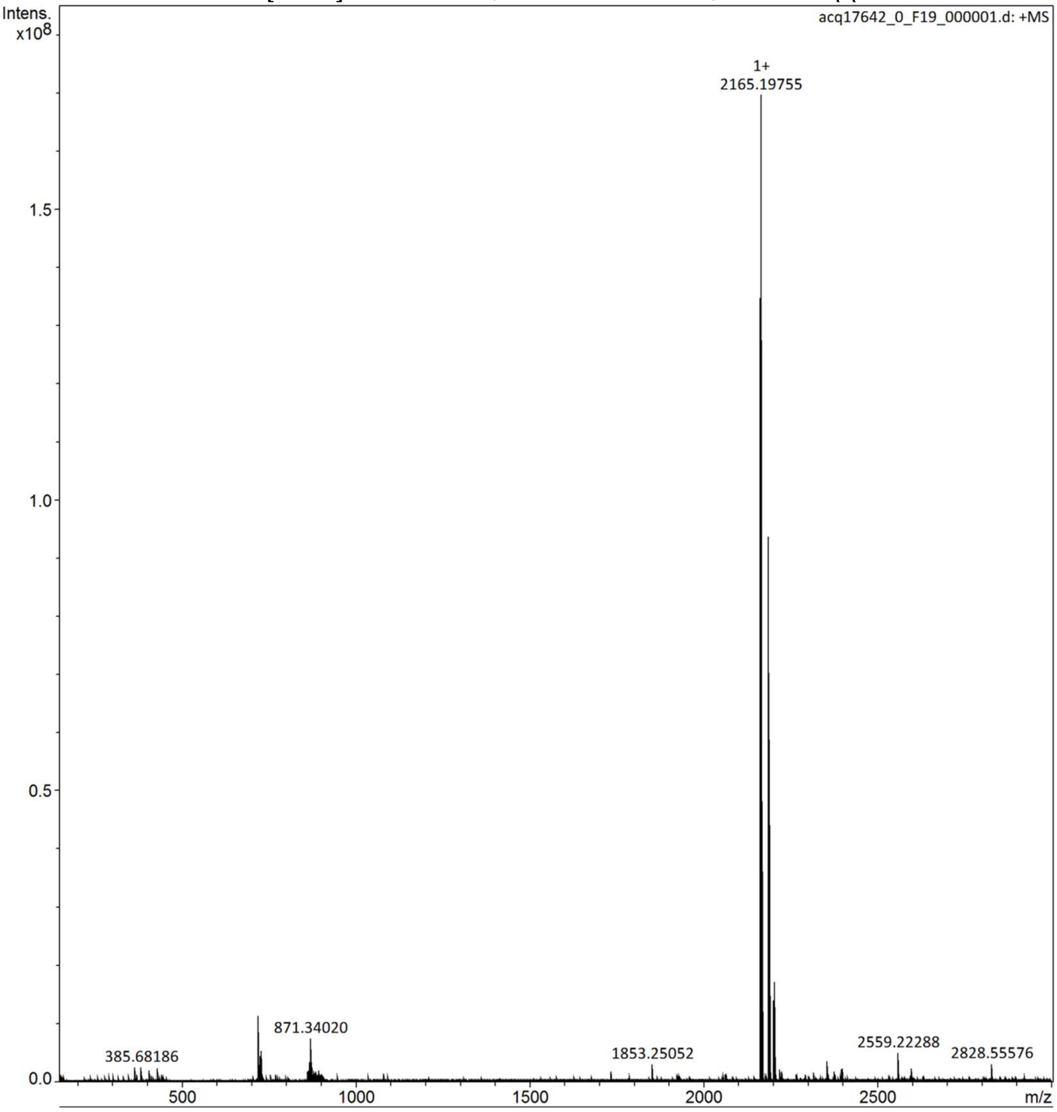


**HPLC:** Charge: +7. $t_R$ = 6.422 min, purity 100.000%. Gradient: 0-60% B during 10 min. B = 95% MeCN + 0.1% TFA.

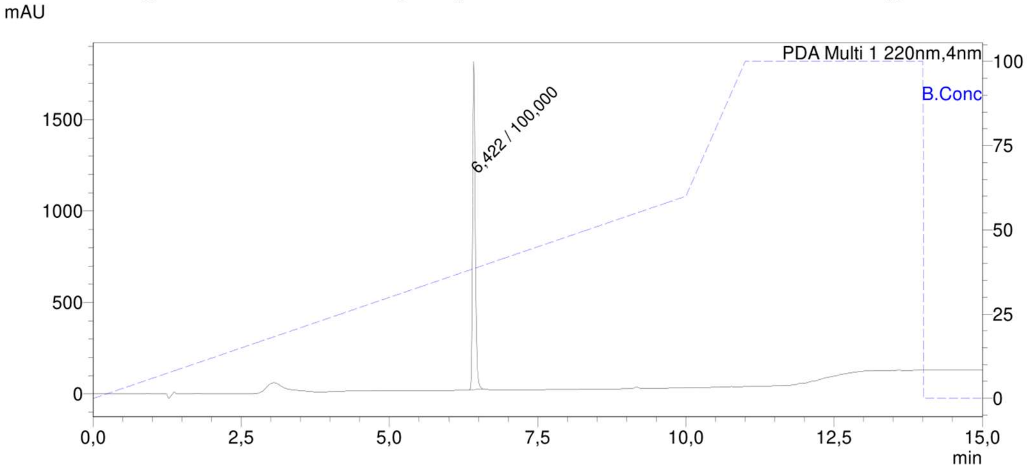

**Compound 12**

**HRMS:** calculated for $[M+H]^{1+}$ 2297.27649, found 2297.27721; ΔM = 0.3 ppm.

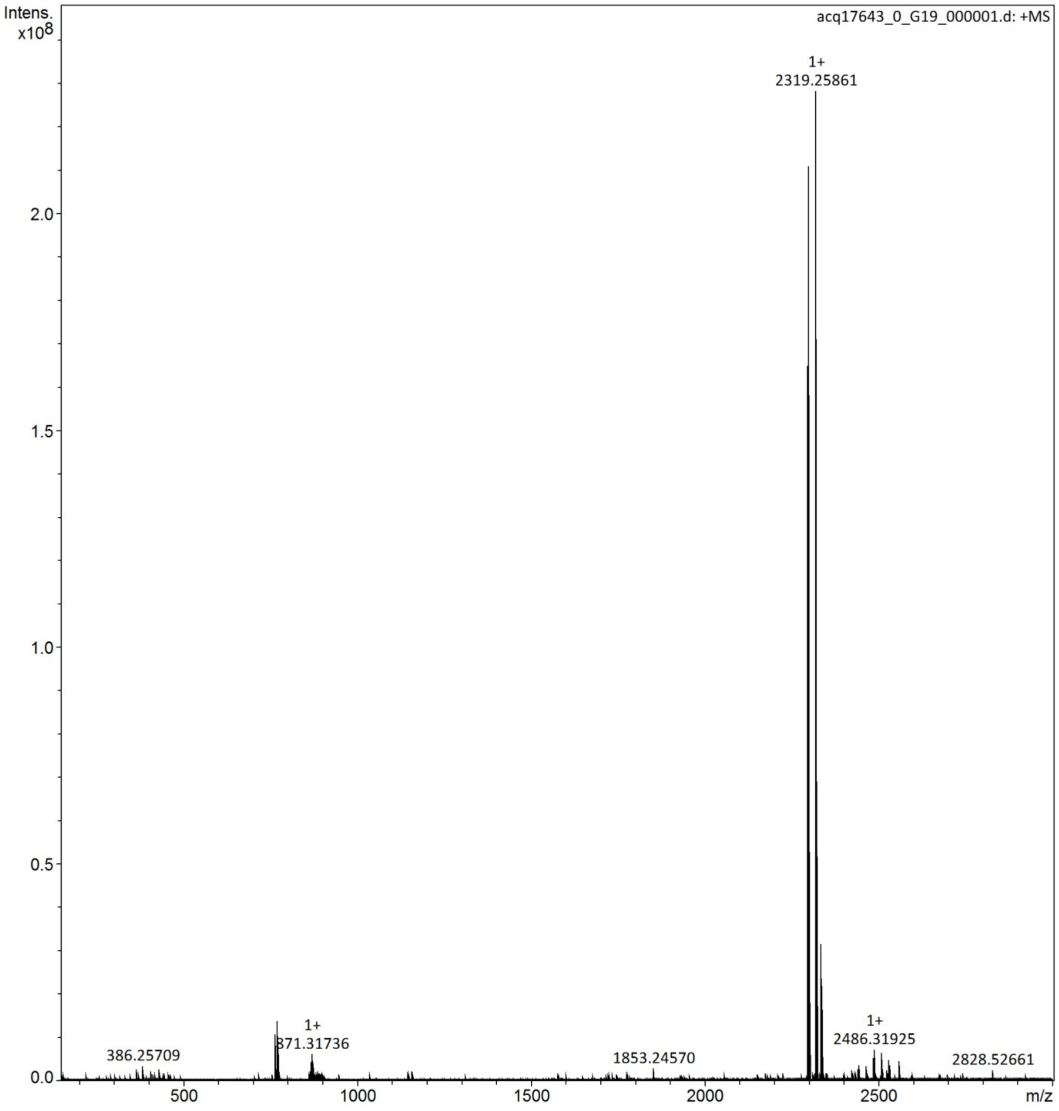


**HPLC:** Charge: +7. $t_R$ = 6.504 min, purity 100.000%. Gradient: 0-60% B during 10 min. B = 95% MeCN + 0.1% TFA.

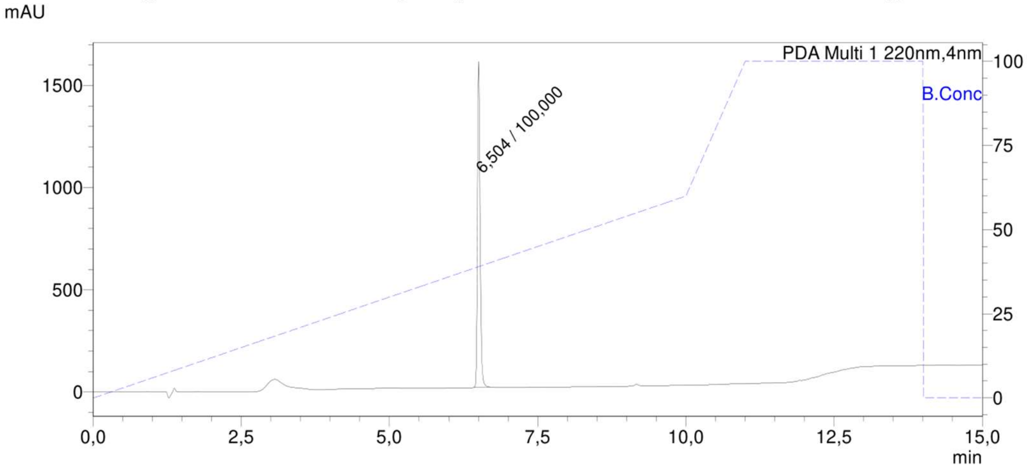

## Compound 13

**HRMS:** calculated for $[M+H]^{1+}$ 2429.35514, found 2429.35620; ΔM = 0.4 ppm.

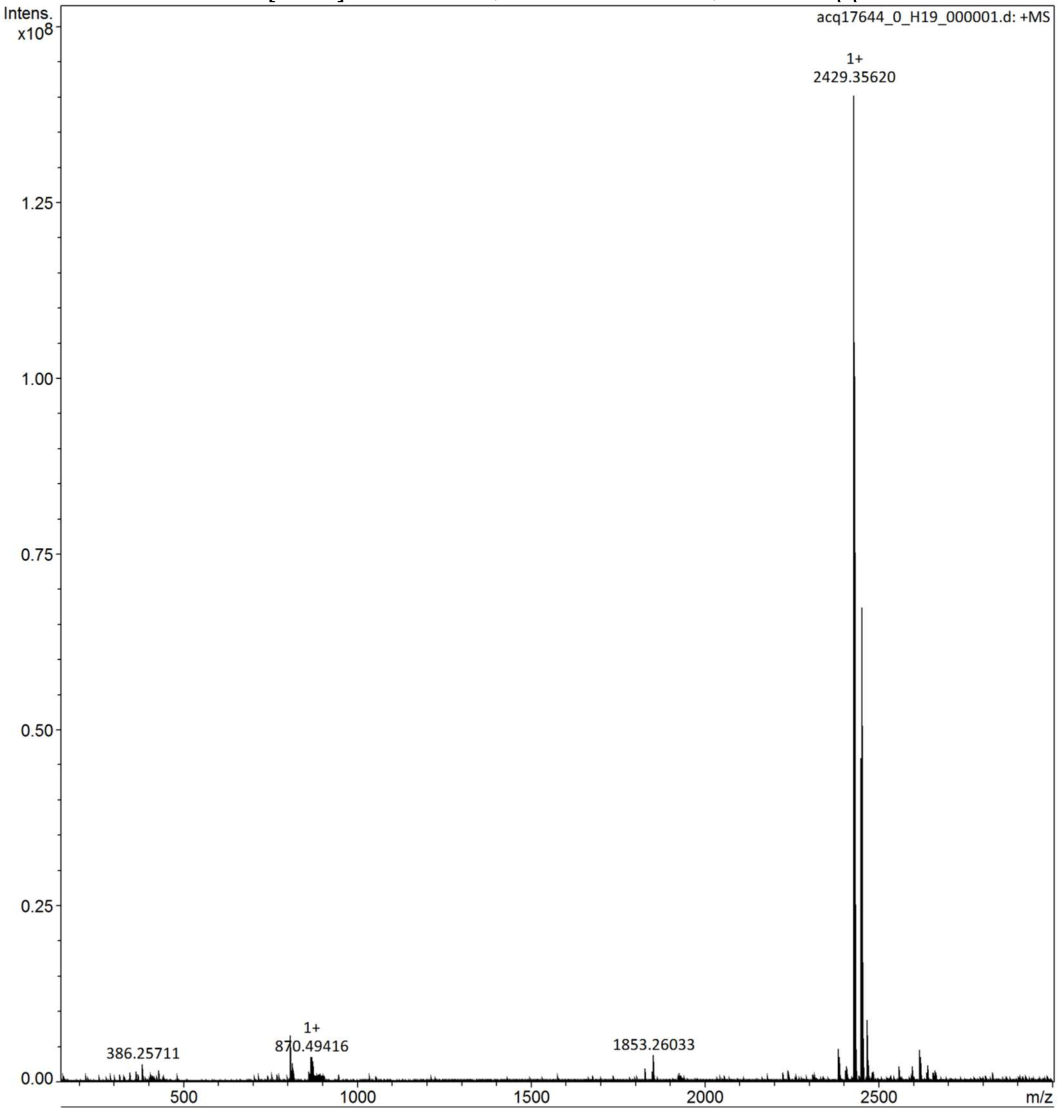


**HPLC:** Charge: +7. $t_R$ = 6.600 min, purity 98.906%. Gradient: 0-60% B during 10 min. B = 95% MeCN + 0.1% TFA.

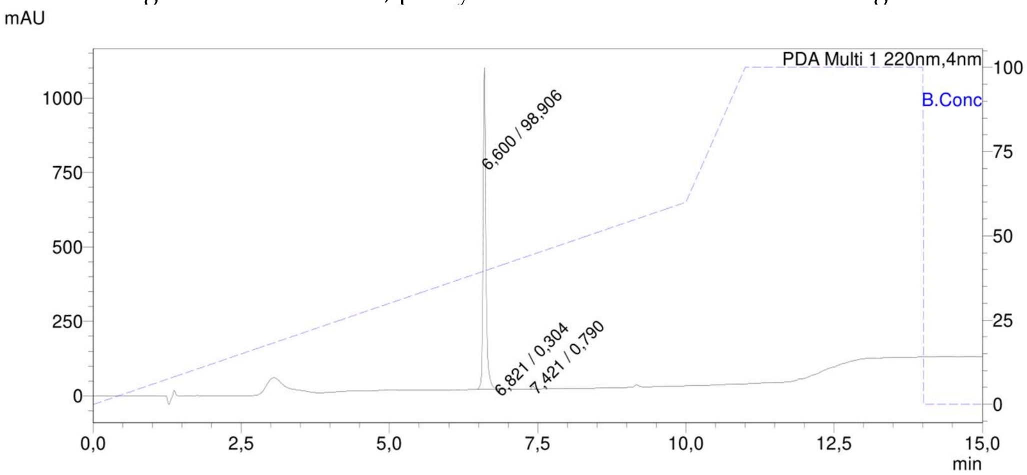

**Compound 14**

**HRMS:** calculated for $[M+H]^{1+}$ 2603.48073, found 2603.47676; ΔM = 1.5 ppm.

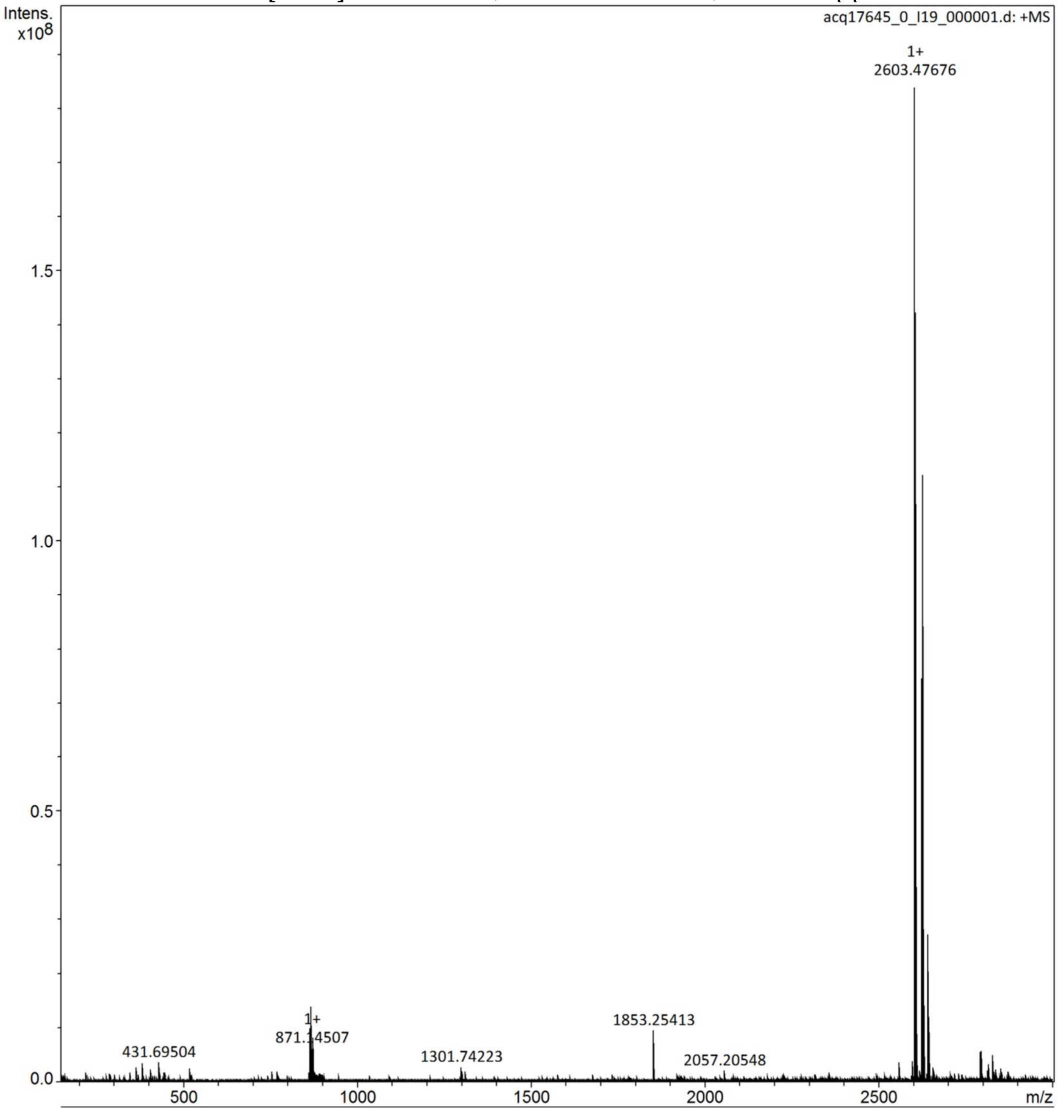


**HPLC:** Charge: +7. $t_R$ = 6.679 min, purity 100.000%. Gradient: 0-60% B during 10 min. B = 95% MeCN + 0.1% TFA.

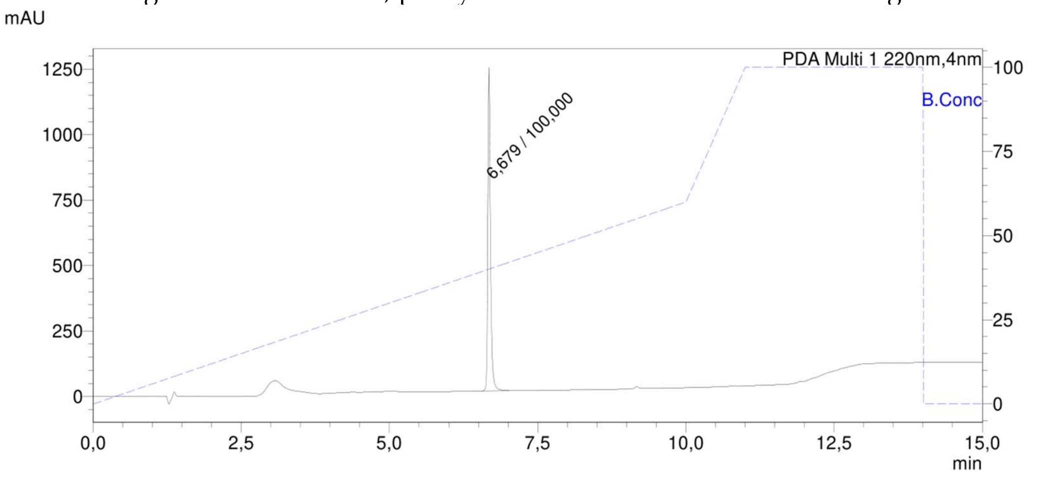

**Compound 15**

**HRMS:** calculated for $[M+H]^{1+}$ 2064.15017, found 2064.15027; ΔM = 0.0 ppm.

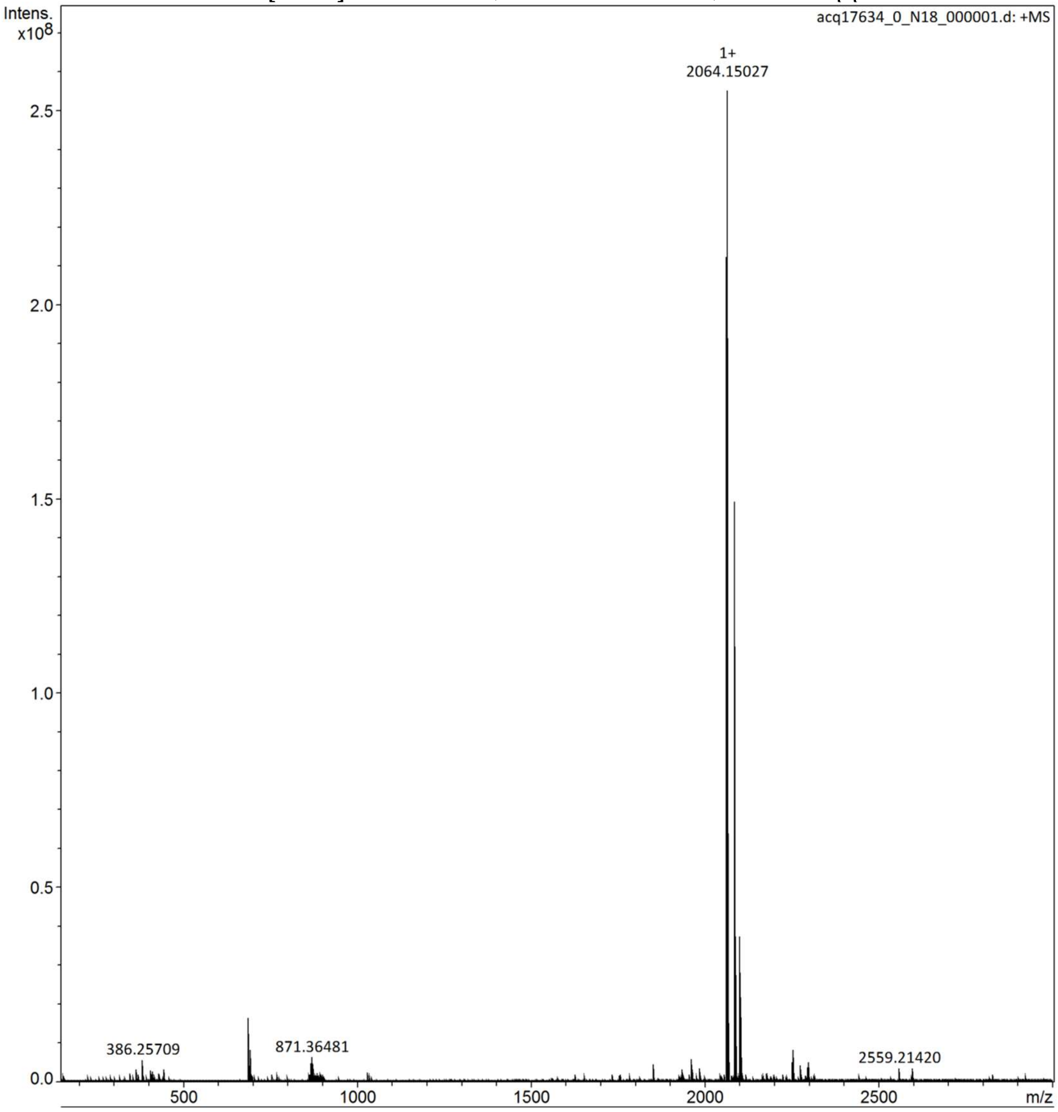


**HPLC:** Charge: +7. $t_R$ = 6.460 min, purity 97.165%. Gradient: 0-60% B during 10 min. B = 95% MeCN + 0.1% TFA.

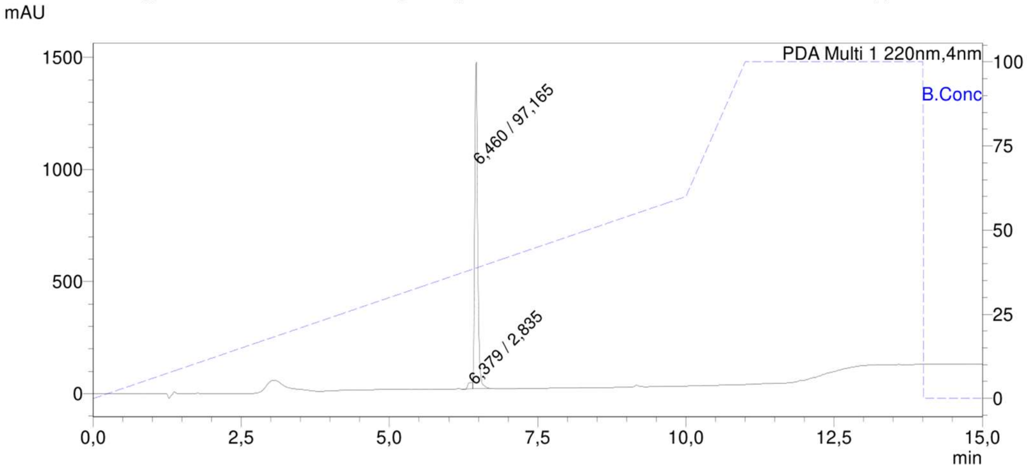

**Compound 16**

**HRMS:** calculated for $[M+H]^{1+}$ 2152.20260, found 2152.20225; ΔM = 0.2 ppm.

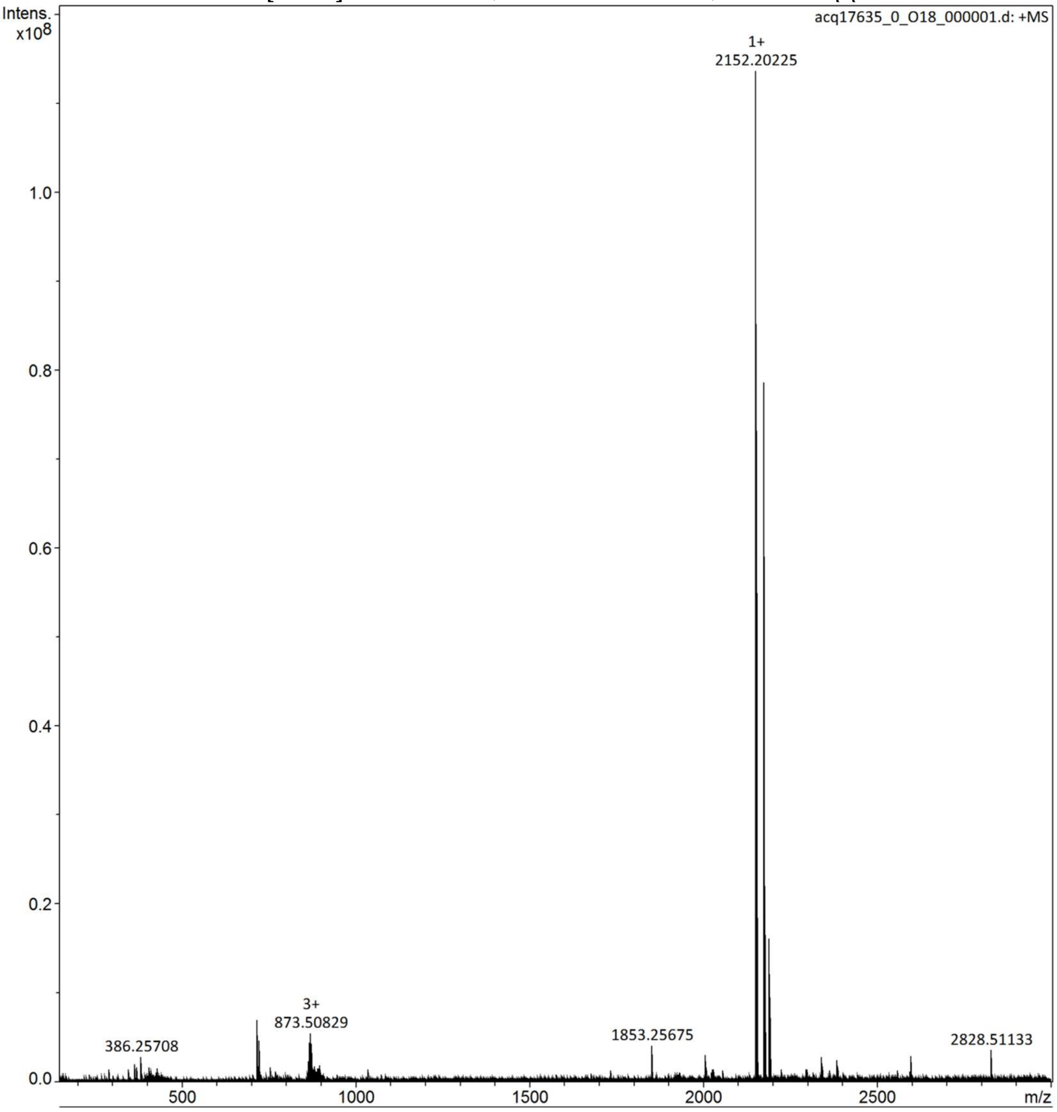


**HPLC:** Charge: +7. $t_R$ = 6.521 min, purity 97.108%. Gradient: 0-60% B during 10 min. B = 95% MeCN + 0.1% TFA.

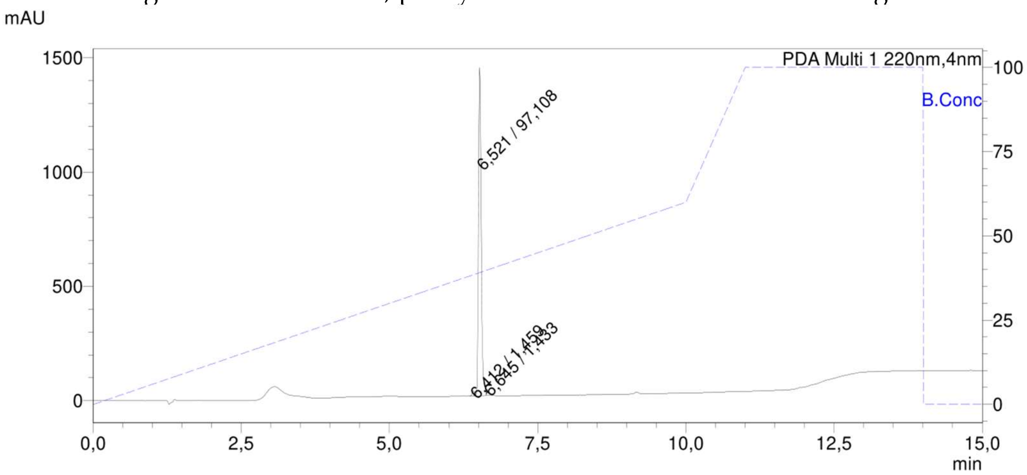

**Compound 17**

**HRMS:** calculated for [M+H]$^{1+}$ 2240.25503, found 2240.25527; ΔM = 0.1 ppm.

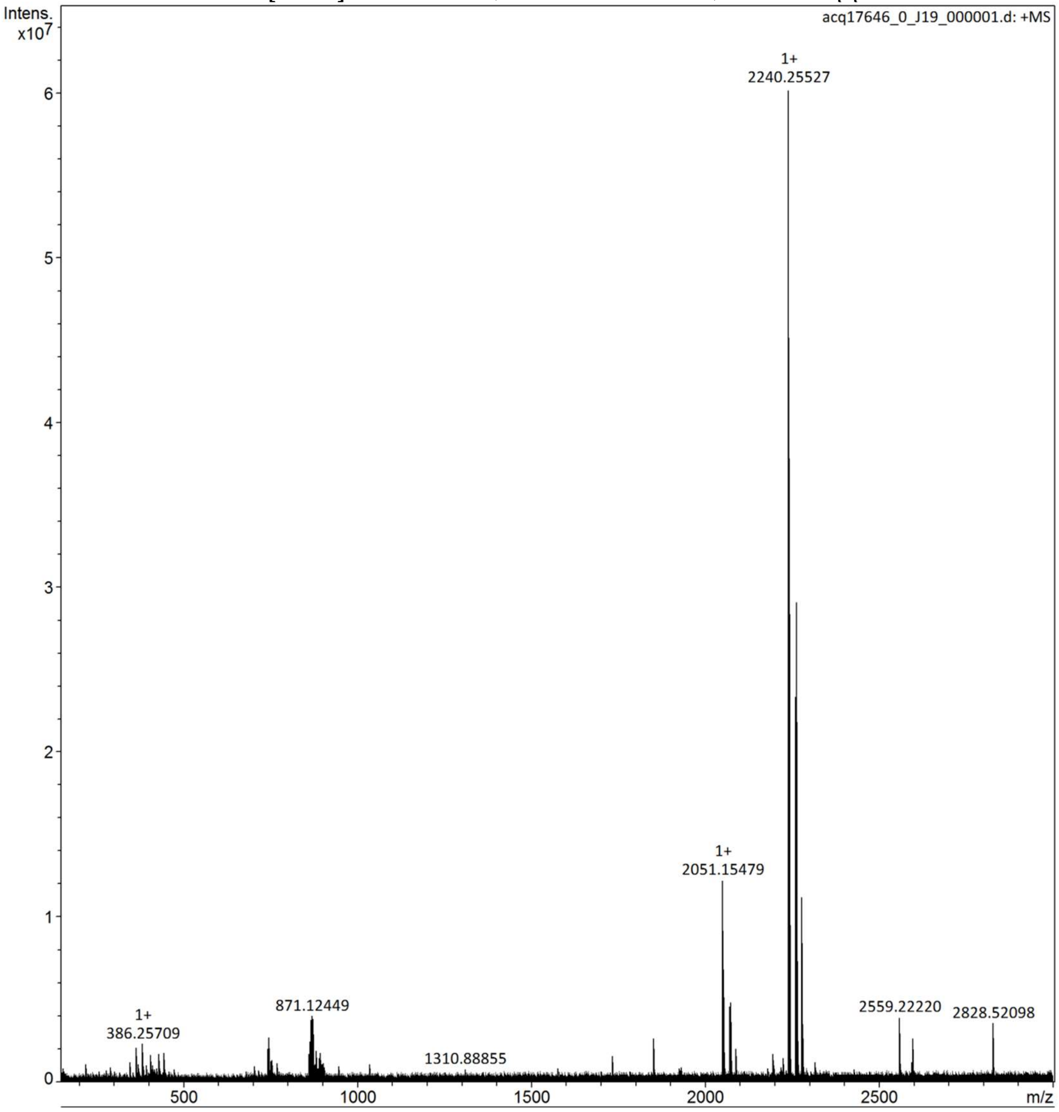


**HPLC:** Charge: +7. $t_R$ = 6.587 min, purity 100.000%. Gradient: 0-60% B during 10 min. B = 95% MeCN + 0.1% TFA.

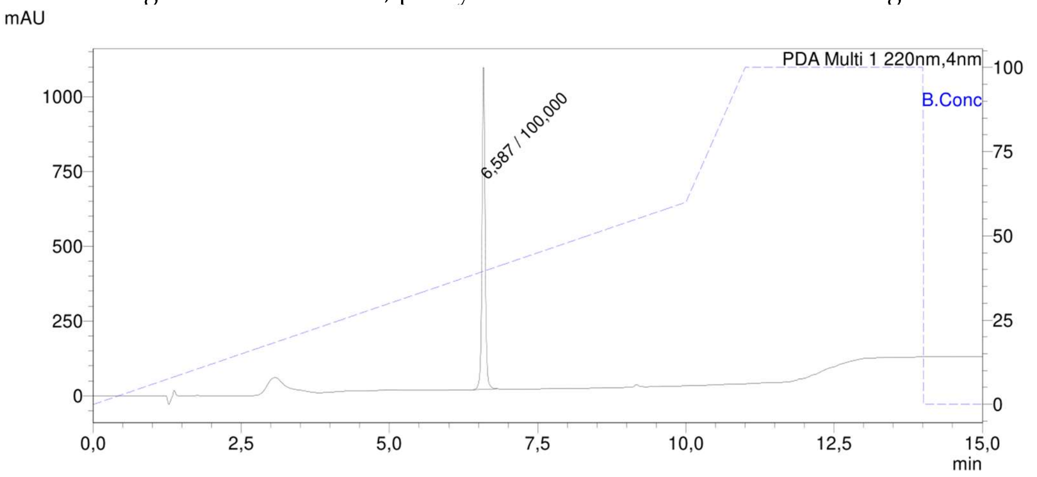

**Compound 18**

**HRMS:** calculated for [M+H]$^{1+}$ 2356.33876, found 2356.33810; ΔM = 0.3 ppm.

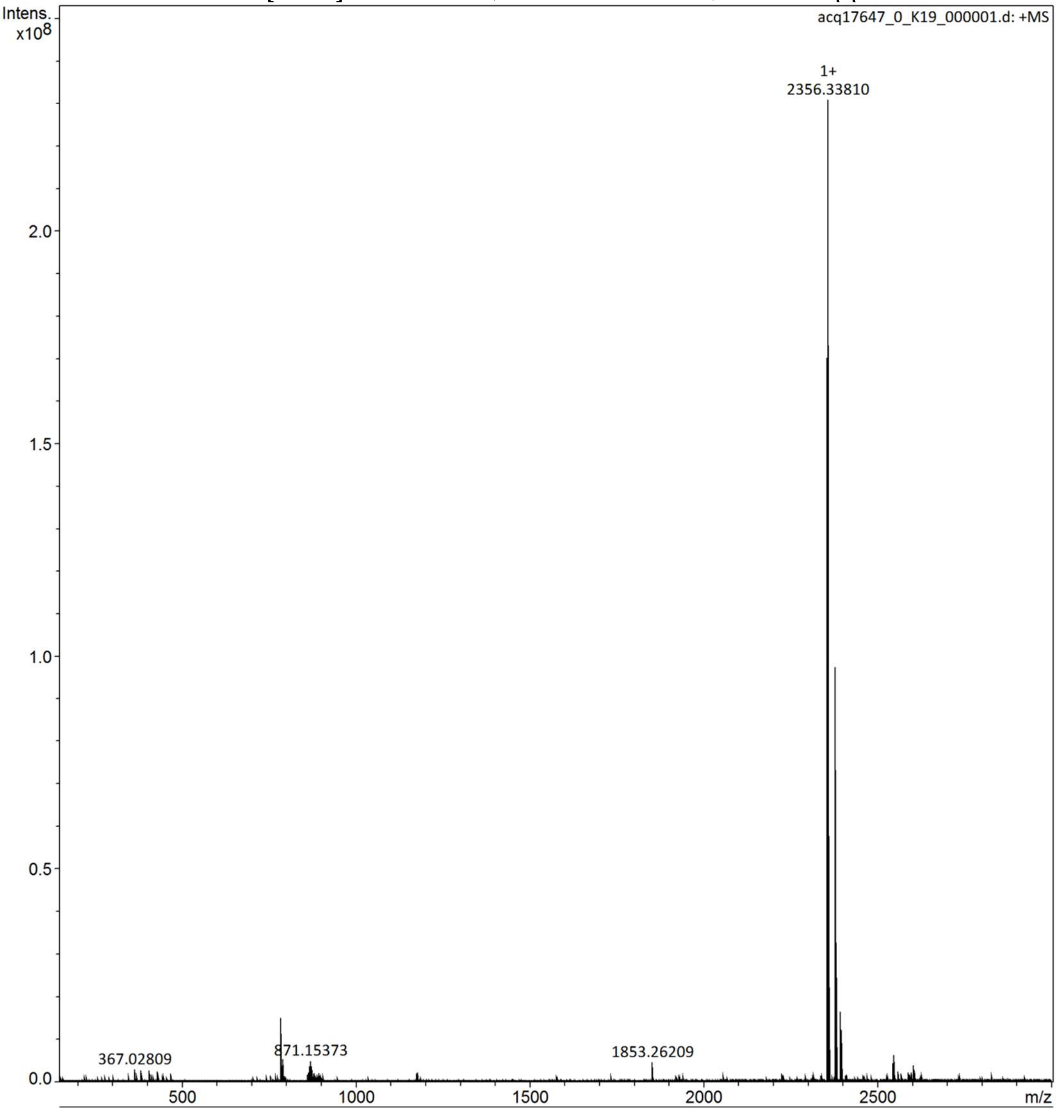


**HPLC:** Charge: +7. $t_R$ = 6.631 min, purity 100.000%. Gradient: 0-60% B during 10 min. B = 95% MeCN + 0.1% TFA.

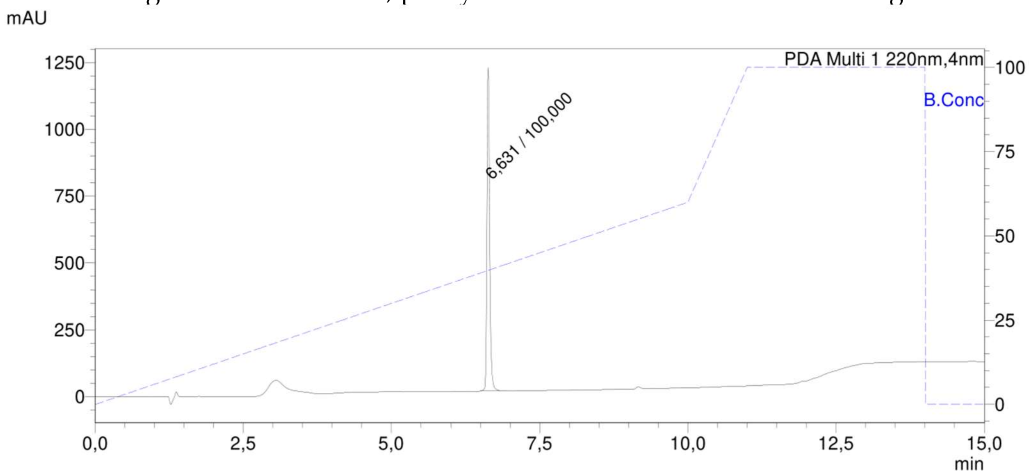

**HepG2 $IC_{50}$ curves**

Curves only drawn for compounds diplaying $IC_{50}$ < 1280 μg/mL, i.e., **1**, **6-10**, **12**, **14-16**.

**Compound 1**

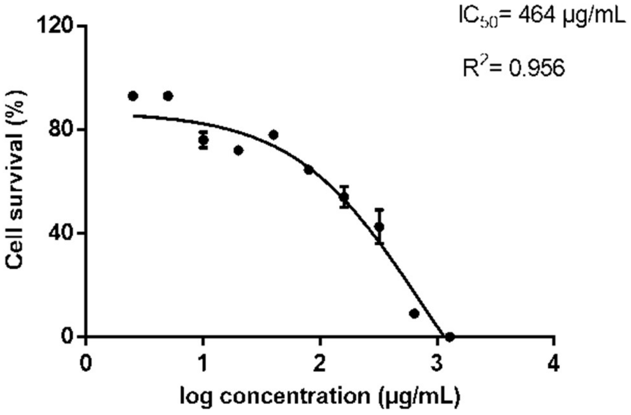


**Compound 6**

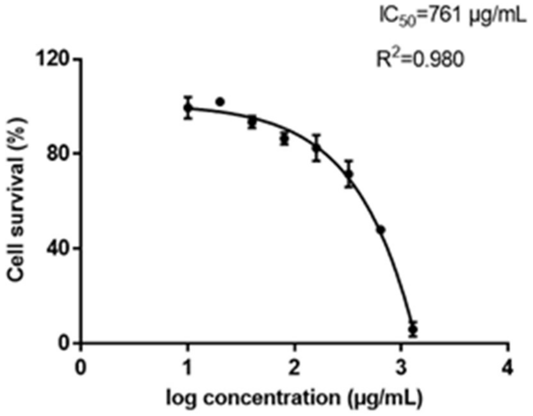


**Compound 7**

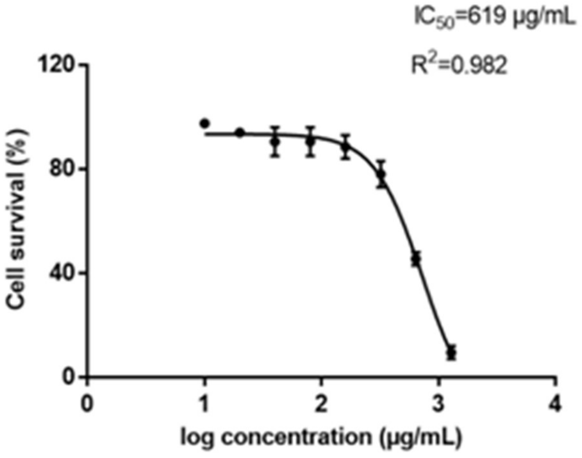


**Compound 8**

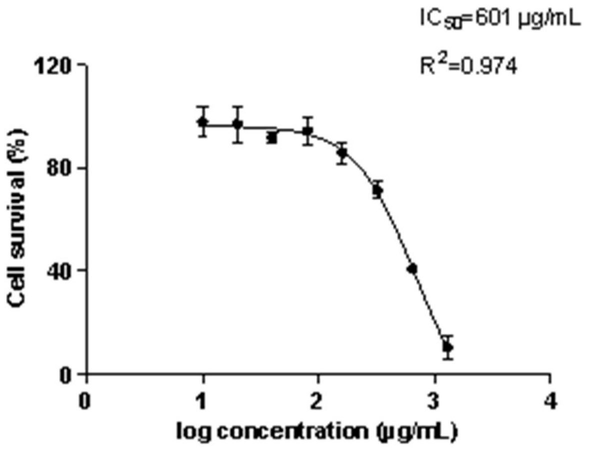


**Compound 9**

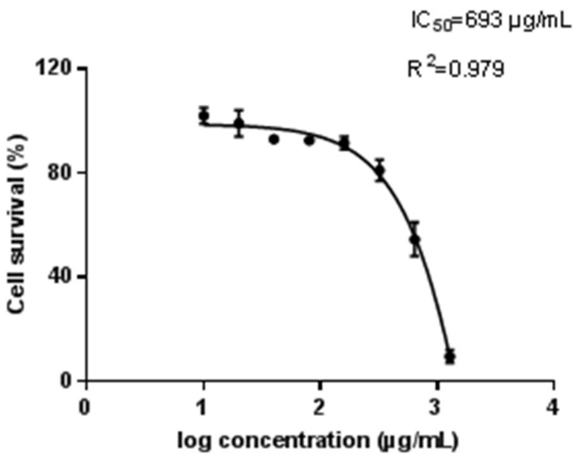


**Compound 10**

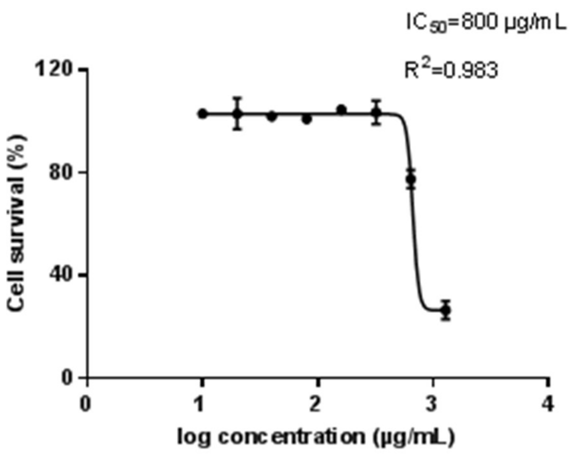

**Compound 12**

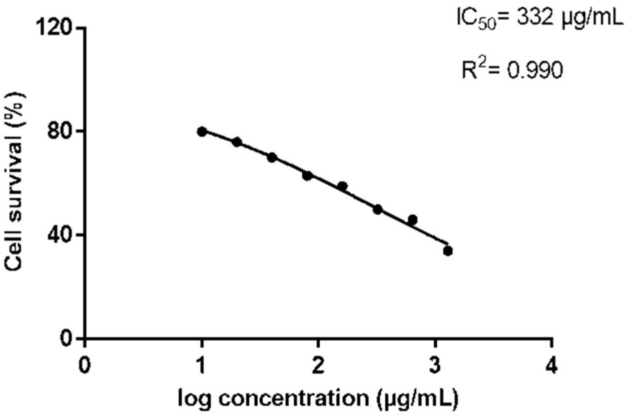


**Compound 14**

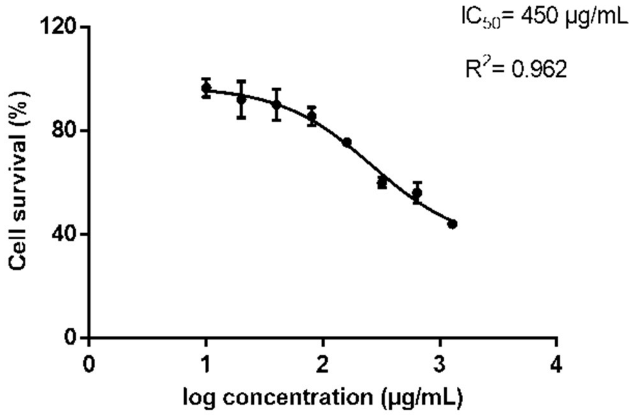


**Compound 15**

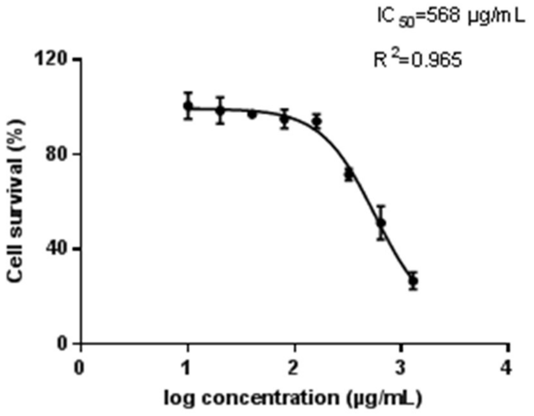


**Compound 16**

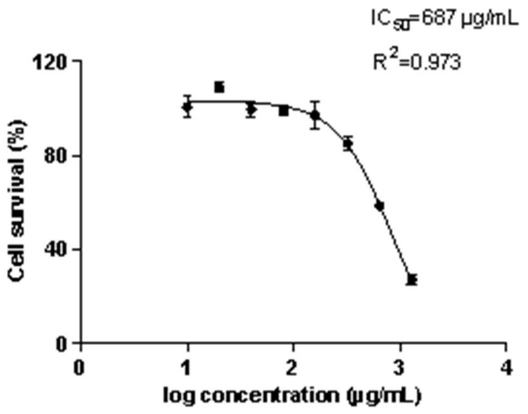

Supplement: Supplementary file 1 [file ijms-22-07041-s001.zip › ijms-1264540-supplementary.pdf]
